# Synonymous and non-synonymous variants at splice junctions can disrupt splicing and are frequently linked to disease associated loss of function genes

**DOI:** 10.1186/s12864-025-12466-0

**Published:** 2025-12-23

**Authors:** Sruthi Srinivasan, Swetha Subramanian, Hui Zhou, Anselm Hoppmann, Patrick Metzger, Melanie Boerries, Senthilkumar Ramamoorthy

**Affiliations:** 1 Division of Pediatric Hematology and Oncology, Department of Pediatrics and Adolescent Medicine, Faculty of Medicine, Medical Center-University of Freiburg, University of Freiburg, Freiburg, Germany; 2 Institute of Medical Bioinformatics and Systems Medicine (IBSM), Faculty of Medicine, Medical Center- University of Freiburg, University of Freiburg, Freiburg, Germany; 3 German Cancer Consortium (DKTK), Partner site Freiburg, a partnership between DKFZ and Medical Center - University of Freiburg, Freiburg, Germany

**Keywords:** Splice junction, Exonic splice variants, Splicing, Canonical splice sites, Silent variants, Missense variants, COSMIC, Cancer genomics, Variant classification

## Abstract

**Background:**

RNA splicing facilitated by the spliceosome complex, relies on specific motifs at exon-intron junctions of pre-mRNAs to generate mature mRNAs. Mutations in splice junctions can disrupt splicing, potentially leading to premature protein truncation. Nucleotides within the exonic component of the junction are also essential for splicing. Evaluation of silent and missense variants in the exonic splice junction on RNA splicing is essential to investigate the significance of such variants in disease pathogenesis.

**Methods:**

We analyzed cancer-associated silent and missense variants reported in the COSMIC database that are located within three nucleotides of splice donor and acceptor sites. We examined the prevalence of these variants in genes for which loss of function is a known mechanism of disease development. We also studied the performance of splicing impact prediction tools and evaluated their clinical relevance as well as their alignment with experimentally validated splicing outcomes.

**Results:**

Nucleotide composition analysis revealed a high preference for the nucleotide G at the donor 1 (d1) and acceptor (a1) positions, 87% and 69%, respectively. We observed a high prevalence of G > A and G > T variants at d1 and a1 positions. Interestingly, 66% to 86% of the identified variants at these positions are missense mutations, with G > T variants being specific for this type of mutation. Evolutionary conservation analysis indicates high nucleotide conservation for these positions at donor and acceptor sites, highlighting their importance at the nucleotide level. The frequently occurring variants are associated with tumor suppressor genes, and 58 of the top 100 genes have LOEUF scores below 1, indicating low tolerance to protein truncation. In contrast, such genes are rarely observed among population variants.

**Conclusions:**

Our data driven computational study emphasizes the importance of evaluating silent and missense variants at splice junctions to understand their impact on RNA splicing. These variants may have a neutral effect on protein function. However, evaluating their effect at the RNA level is essential to understanding the significance of these variants in disease pathogenesis. This is particularly important for genes in which loss of function is the mechanism of disease development.

**Supplementary Information:**

The online version contains supplementary material available at 10.1186/s12864-025-12466-0.

## Background

Recent advances in sequencing technologies have transformed our understanding of disease etiology by uncovering numerous mutations associated with cancer and various genetic disorders. Experimental characterization of variants to determine their effects on protein/RNA function is crucial for understanding disease pathogenesis. However, this is often challenging due to the large volume of data from genomic studies. To address this challenge, variants are evaluated based on their presence in the healthy population, co-segregation, evolutionary conservation, and computational predictions of their effects on protein structure and function [1]. In addition to variants affecting protein function, those in the non-coding genome can influence gene expression, RNA stability, structure, and splicing.

In the eukaryotic genome, protein-coding exons are interspersed with non-coding introns. The intronic regions must be precisely removed from the pre-mRNA, and the exons are joined together while maintaining the reading frame of the protein-coding region. The splicing of pre-mRNA is carried out by spliceosome complex consisting of five major small nuclear ribonucleoproteins (snRNPs) [2–4]. Accurate recognition of the nucleotide signature at the splice junction by the spliceosome complex is essential for proper splicing of pre-mRNA [5]. The RNA components of the snRNPs recognize the splice sites at the exon-intron boundaries and mediate the assembly of the spliceosome complex members and the precise joining of the exons. The 5’ and 3’ splice sites at the exon-intron junction consists of a specific consensus nucleotide pattern essential for proper splicing. The intronic region of the splice junction contains a dinucleotide GU motif at the 5’ donor site, while the 3’ acceptor site contains an AG motif. Additionally, a few nucleotides around these motifs in both intronic and exonic regions have a conserved consensus nucleotide pattern. The exonic component of donor regions contains a [C/A]AG motif, while the exonic component of acceptor sites contains a GU motif [6–8]. In addition, exons and introns contain enhancer and suppressor elements that regulate the proper landing and assembly of spliceosome complexes. Variants disrupting splicing sites are critical drivers of cancer gene mutations, particularly in genes where haploinsufficiency drives disease development [6, 9–12]. Mutations in the canonical splice sites lead to exon skipping. The impact of variants at splice junctions is evaluated using a combination of computational methods and experimental validation [13]. The impact of a variant on the associated gene splicing is evaluated using functional assays, such as minigene reporter assays, RNA-seq and targeted RT-PCR. Large-scale screening approaches, such as the multiplexed functional assay of splicing (MFASS) with Sort-seq, are used to study the variant impact on splicing [14]. Computational prediction tools are effective in identifying variants that disrupt canonical splice sites or create new cryptic sites, due to the presence of consensus motifs at the splice junction [15–21]. However, the impact of the variants in the exonic regions on splicing is often overlooked. These variants are primarily assessed and documented for their effect on protein function. In addition to the core dinucleotide in the intron of donor and acceptor sites, a significant proportion of variants that affect splicing extend beyond the core splice region [22]. The population variants are rare around the splice junction while disease-causing variants are frequently observed [23, 24]. A massively parallel splicing assay (MaPSy) study of disease-causing exonic variants revealed that approximately 10% of the variants result in altered splicing [25]. Exonic variants associated with rare diseases that disrupt normal splicing were identified in a large cohort of samples from the 100,000 genomes project [26]. Recent ACMG guidelines recommend assessing the effect of variants on splicing for up to 3 and 1 nucleotides of the exonic regions of the donor and acceptor arms, respectively [27]. Synonymous mutations have been the focus of several genome-wide studies to assess the impact of exonic variants on splicing and RNA stability due to their neutral effect on protein function [28–30]. However, gene centric experimental studies have shown that both silent and missense mutations significantly affect splicing, particularly at the last three nucleotide positions on acceptors and donors [17, 31–33]. A study focusing on disease-associated missense and silent variants in COL4A5 revealed that 17 of the 20 evaluated variants disrupt splicing from the last nucleotide of the coding exons [34]. Most variant annotation tools and databases primarily assess and report exonic variants impact on protein function. A detailed evaluation of exonic variants in the context of splicing would have significant implications for understanding disease outcomes. In the present study, we conducted an in-depth analysis of cancer associated silent and missense variants at the canonical splice junction, focusing three nucleotides of exonic region adjacent to acceptor and donor sites. We have also sought to determine the prevalence of exonic variants in genes where loss of function is a mechanism of disease development, using both population and cancer datasets.

## Methods

The human canonical protein-coding genes list was obtained from the Gencode consortium. A total of 19698 canonical protein-coding transcripts, comprising 206147 exons, were extracted from the human genome assembly (v43, GRCh38) for the analysis. To preserve only the protein-coding exons, the 5’ and 3’ untranslated regions (UTRs) of the transcripts were excluded. The orientation of the transcripts in the reference assembly was considered while defining the donor and acceptor splice sites. The last three nucleotides of the donor site and the first three nucleotides of the acceptor site were extracted from the non-redundant list of exons using bedtools. Nucleotide positions − 1, -2, and − 3 of the donor sites are denoted as d1, d2, and d3, while positions + 1, +2, and + 3 of the acceptor sites are denoted as a1, a2, and a3, respectively.

Missense and silent variants reported in the COSMIC database were extracted and overlapped with the donor and acceptor sites using the bedtools intersect function. The annotation of unique silent and missense variants at the donor and acceptor sites was performed using ANNOVAR and VEP (v114) tools [35, 36]. ANNOVAR provided annotations from several genomic databases, including RefGene, gnomAD, ICGC, ClinVar, dbNSFP (v47a), and COSMIC. The COSMIC variants were filtered using a gnomAD allelic frequency cutoff of 0.1% in order to exclude those that are commonly found in the population. The Genome Aggregate Database (gnomAD) represents an extensive repository of populations variant data and provides valuable insights into the interpretation of novel genes, especially for rare diseases and to study biological effects of genetic variation [37]. The silent and missense population variants at the splice junctions from the gnomAD database were extracted as described for the COSMIC datasets. The gnomAD datasets compiled by ANNOVAR were used to identify population variants at the exonic components of splice junctions (v4.1). A minor allele frequency (MAF) threshold was used to categorize population variants as very rare or common: <0.1% for very rare variants and ≥ 0.1% for rare and common variants.

The clinical significance of the variants identified in the COSMIC and gnomAD datasets was investigated using the ClinVar database, a large collection of publicly curated records that aid in understanding the pathogenicity of variants [38]. ClinVar (v240917) variant annotation was performed using the ANNOVAR tool. To infer the evolutionary conservation of the variants, we used the PhastCons score. PhastCons employs a Markov model to determine the likely contribution of individual nucleotides to conserved elements [39]. PhastCons20 is derived from the alignment of 20 vertebrate species. The score indicates the probability of conservation at the nucleotide level and ranges from 0 to 1. The Loss-of-Function (LoF) mutations can have deleterious effects or no effect on protein function. Genes with pathogenic LoF mutations are found in low numbers in natural populations due to the effects of purifying selection [40]. The loss-of-function (LoF) impact of the variant on the associated genes was assessed using the Loss-of-Function Observed/Expected Upper Bound Fraction (LOEUF) score [41]. This metric evaluates the tolerance of genes to protein-truncating variants based on their prevalence in the healthy population. A LOEUF score below 0.33 is considered to have a significant impact on gene function, while a score above 1 is generally associated with a relatively neutral effect. Scores between 0.33 and 1 are considered to have a moderate to less severe effect. The incidence of each individual variant in the COSMIC cancer dataset was obtained using the ANNOVAR tool. The list of tumor suppressors, oncogenes, and Cancer Gene Census (CGC) genes was obtained from the COSMIC database [42]. The CGC is the largest collection of genes associated with cancer development. It includes genes with mutations that cause cancer, as well as genes with a predisposition to develop cancer and inherited in the population. The significance of the overlap was calculated by hypergeometric test implemented in the R function “phyper”. The statistical significance of genes with low LOEUF scores in the COSMIC and gnomAD datasets was assessed using a two-sided chi-square test implemented in the R function “chisq.test”. A Kruskal–Wallis rank-sum test was conducted using R to assess variant count differences among cancer tissue types. To determine the top genes with the most variants at the donor and acceptor sites, we normalized the number of variants per exon at each splice site. To avoid variability due to the number of exons per gene, we used exon count as the normalizing factor.

The impact of the variants on splicing was assessed by employing *in silico* prediction programs SpliceAI (v1.3), Pangolin (v1.0.1), CADD (v1.7), SPiP (v2.1) and MaxEntScan (v2004.04.21-4) [16, 19–21, 43]. The variant annotation tools VEP was used to derive the pre-computed SpliceAI score and to calculate MaxEntScan score. While evaluating the variants, we applied a MaxEntScan difference score greater than 0 as the cut-off to visualize potential splice site impacts. In addition to this, the alternate (alt) MaxEntScan score can be used to stratify the variants as high (< 6.2), moderate (6.2–8.5), or low (> 8.5) potential to disrupt the native splice site [44]. CADD (v1.7) and Pangolin scores were obtained from the source. We obtained a total of 1749 experimentally validated splice-altering variants and 401 variants that do not affect splicing. Most of the variants were obtained from SpliceVarDB, MutSpliceDB and the datasets curated by Li et al. [45–47]. The receiver operating characteristic (ROC) curve analysis was performed using the function “roc” from the R package pROC. We looked up for the precalculated 49 human tissues specific aberrant splicing effect scores generated by AbSplice [48]. The exonic splice junction variants of TP53 were visualized using ProteinPaint [49]. For each variant, the highest splicing effect score among the 49 tissues was used to assess the impact of the variant. The experimentally evaluated splice impacting donor and acceptor site variants reported in SpliceVarDB were overlapped to all, COSMIC and gnomAD sites and visualized as a river plot using the R package “ggsankey”. The same approach was applied to identify overlaps between the variants and the ClinVar datasets.

## Results

**Fig. 1:**
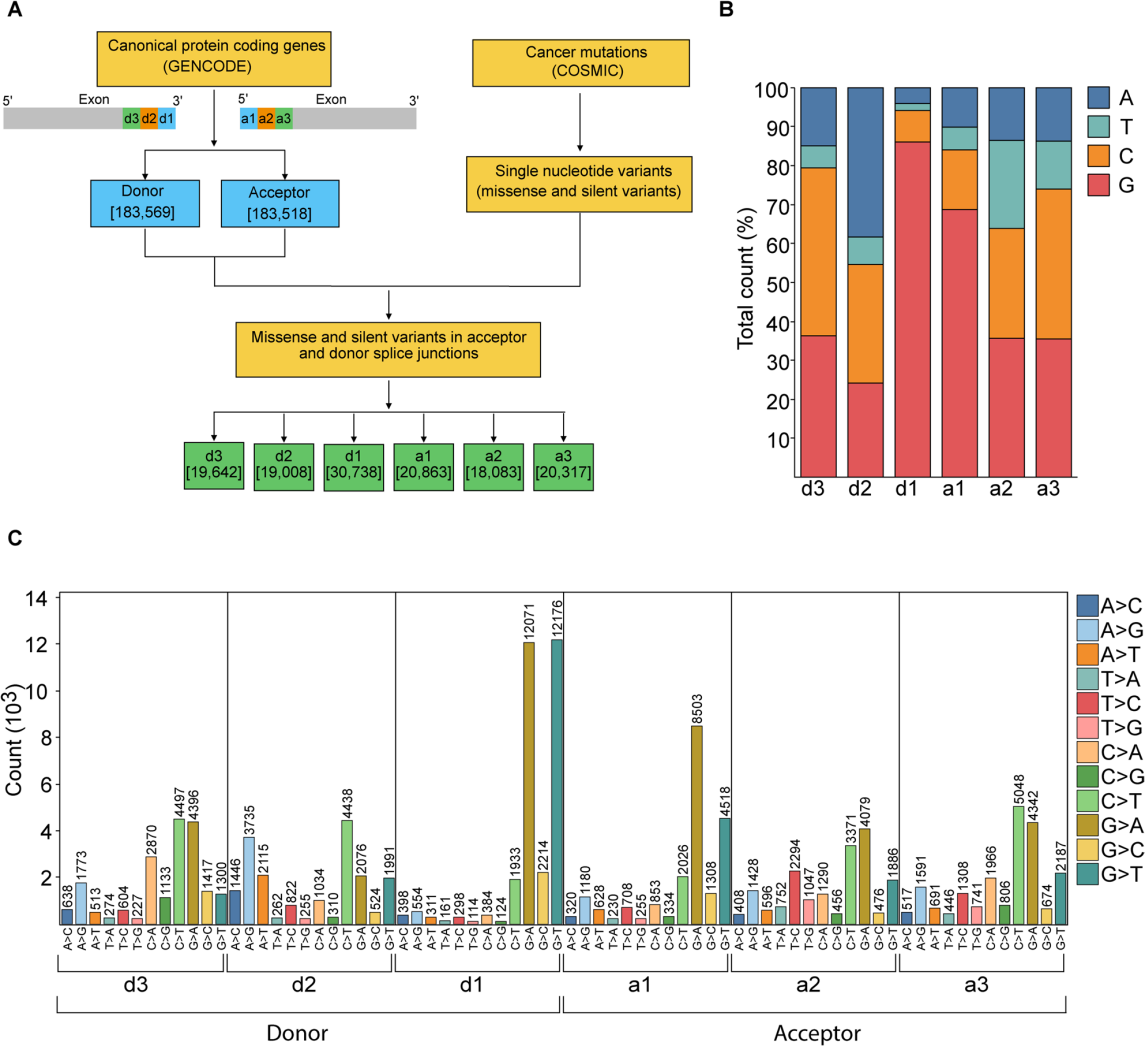
Preferential alteration of certain nucleotides in silent and missense variants at the splice junction. **(A) **Workflow for identifying cancer-associated somatic silent and missense variants at the splice junction. The donor and acceptor sites were obtained from the GENCODE. Silent and missense variants from the COSMIC database overlapping the three exonic nucleotides at the donor splice site (positions d1, d2, and d3) and the three exonic nucleotides at the acceptor splice site (positions a1, a2, and a3) were identified. **(B) **The reference nucleotide composition of sites with silent or missense variants in the COSMIC database. Bars are color-coded by the reference nucleotide base. **(C) **The COSMIC nucleotide variant signature in the exonic donor and acceptor sites. The x-axis represents the reference and variant nucleotides while the y-axis shows the total abundance of each variant. Bars are color-coded according to the type of nucleotide change

Advances in integrating experimental data and computational prediction algorithms have significantly improved the accuracy of human genome annotation [50]. The Gencode consortium provides a high-quality annotation of the human genome. A total of 206,147 exons in canonical protein-coding genes are reported in the Gencode database (v43, GRCh38). This encompasses 183,518 acceptor and 183,569 donor sites (Fig. 1A). A consensus motif prediction at the splice junction indicated an enrichment of specific nucleotide patterns within the donor and acceptor splice sites (Fig. S1) [31]. A distinct consensus nucleotide pattern, consisting of up to three nucleotides in the donor arm and two nucleotides in the acceptor arm, exists as part of the exons at the splice junctions (Fig. S1). The exons showed enrichment of nucleotides G, A and [C/A] at positions − 1, -2 and − 3 of the donor sites (referred to as d1, d2 and d3, respectively) with frequencies of 80%, 64% and 69%, respectively. At the + 1 and + 2 positions of acceptor arm of the exons, 49% of G and 37% of U were detected, respectively. We evaluated missense and silent mutations at these sites, as well as their potential impact on splicing, in both cancer patients and the healthy population. The cancer datasets were obtained from the Catalogue of Somatic Mutations in Cancer (COSMIC) database (v98). For the comparative analysis, very rare and common variants from the healthy population were obtained from the Genome Aggregation Database (gnomAD) (v4.1) [37, 51]. We identified protein coding silent and missense variants, reported in the COSMIC database, that are located within three nucleotides of the donor and acceptor splice junctions. In the donor sites d1, d2 and d3, we identified a total of 30,738, 19,008 and 19,642 unique SNVs respectively. We also found 20,863, 18,083 and 20,317 sites in the acceptor sites a1, a2 and a3, respectively (Fig. 1A). We observed a preferential alteration of certain nucleotides in the donor and acceptor sites in cancer dataset (Fig. 1B). The guanine (G) nucleotide is most commonly altered at the d1 and a1 sites, accounting for approximately 87% and 69% of their respective distributions. However, it is present in the proportion of 80% and 49% of d1 and a1 sites, respectively, in the protein-coding exons of the human genome (Fig. S1). We observed a different scenario at position d2, where we find 64% of sites with nucleotide A in the overall distribution, while only 38% of cancer-associated sites have nucleotide A. We find high preference for G and C nucleotides in a3, a2, and d3 sites. Subsequent analysis of the nature of the nucleotide changes revealed a higher frequency of G > A and G > T variants at the initial position of the donor splice site (d1) and the initial position of the acceptor splice site (a1) (Fig. 1C, Table S1). Notably, a higher prevalence of G > T mutations emerged, with 12,176 and 4518 sites observed at positions d1 and a1, respectively. Additionally, G > A mutations are prominently featured, adding up to 12,071 and 8503 sites specifically at d1 and a1, respectively. Further examination of the data reveals that positions d2, d3, a2, and a3 exhibit a complex landscape characterized by irregularly distributed mutations. At position d2, we found a higher frequency of C > T and A > G variants. At position a2, we found C > T and G > A variants, which are also the two most frequent types observed at positions a3 and d3. Within these regions, a higher prevalence of C > T mutation is observed covering 4438 sites in d2, 4497 in d3, and 5048 in a3. On the other hand, there are more A > G mutations in d2, covering 3735 sites, which is relatively less prevalent in other sites. A preferential pattern of cancer-associated variants indicates nucleotide-centric function at these sites.

### Missense variants are more abundant at the splice junction

**Fig. 2:**
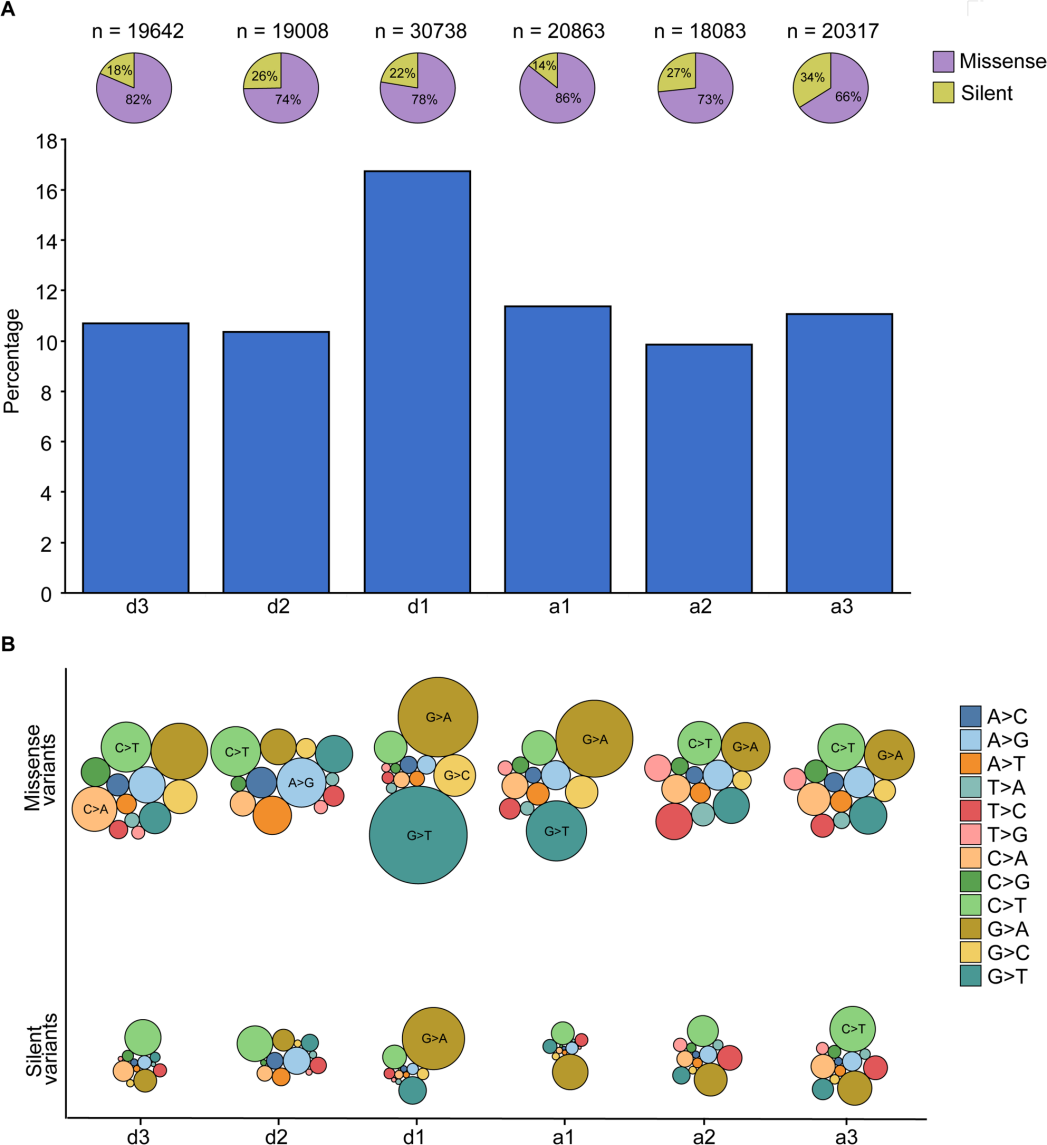
Enrichment of missense variants at the splice junction. **(A) **The total percentage of donor and acceptor sites in human exons having variants in COSMIC database (bars, bottom) and the proportion of silent or missense variants (pie chart, top). **(B) **The bubble plot represents the relative abundance of single nucleotide alterations in the missense and silent variants. The bubbles are color coded according to the type of single nucleotide alterations

Silent variants are often the focus of studies on the effect of protein coding variants on splicing [28, 30, 52, 53]. However, our analysis of silent and missense variants within exonic splice junctions revealed a striking prevalence of missense variants compared to silent variants. Approximately 10 to 17% of human acceptor and donor sites are represented at least once in the COSMIC dataset (Fig. 2A, bottom). Most of these sites (between 66 and 86%) are missense mutations, while a smaller percentage (between 14 and 34%) are silent mutations (Fig. 2A, top). Approximately 17% of donor sites (30738 sites at position d1) are involved in cancer patients. The majority of these sites (78%) are missense mutations, while the remaining 22% are silent mutations (Table S1). Similarly, 11.5% of acceptor sites at position a1 are found in the COSMIC dataset. The majority (86.1%) of these mutations are missense, while the remaining 13.9% are silent. Interestingly, at position d1, we find almost equal numbers of silent and missense variants in the gnomAD common variants, with 54% and 46%, respectively (Table S1).

We also observed a distinct nucleotide pattern among missense and silent variants. A relatively higher rate of G > A nucleotide changes was observed at the d3, a1, a2, and a3 sites in missense variants (Fig. 2B and Table S2). Missense variants with G > T nucleotide changes were frequently observed at the d1 sites. A higher number of G > T (11282) and G > A (7493) mutations were observed at the missense donor site d1. Similarly, 4286 G > T and 7011 G > A mutations were found in missense acceptor site a1. Interestingly, missense variants at the d2 site are enriched for A > G and C > T variants. Among missense variants, C > T variants were relatively more abundant at d2, d3, a2, and a3 sites. We also evaluated the distribution of donor and acceptor site variants across cancer tissues and found no preferential enrichment at specific sites in COSMIC samples (Fig. S2A). Furthermore, a comparative analysis of the total distribution of variants in samples with at least one variant indicated an increased abundance of variants in skin, lung, and endometrial tissues (Fig. S2B). The median occurrence of exonic splice junction variants was 0 across all tissue types. However, we observed a significant difference in variant abundance among tissue types (Kruskal–Wallis test, *p*-value < 2.2E-16; Table S3).

### The silent and missense variants of the splice junctions are often found in the gene with loss of function

**Fig. 3:**
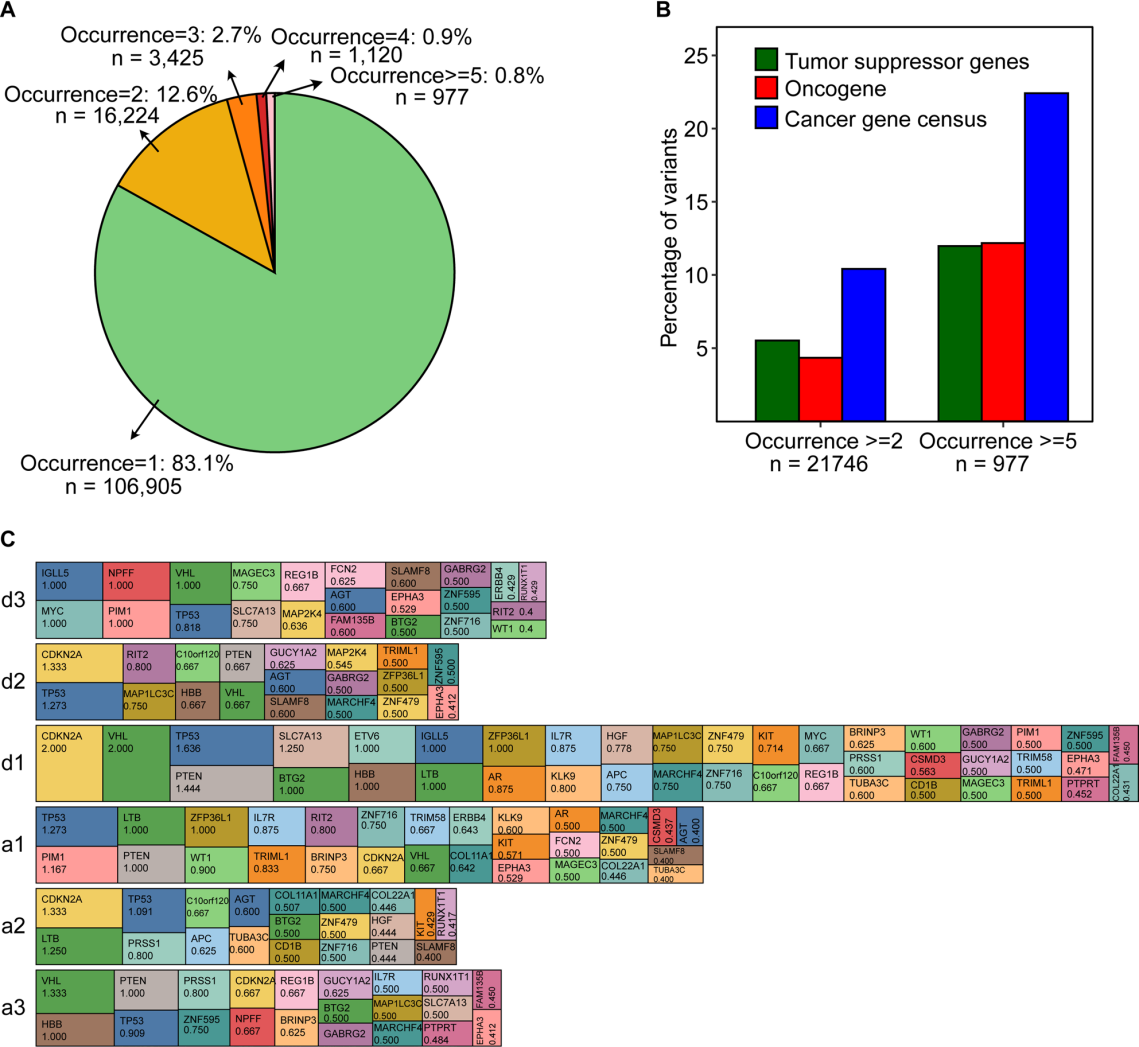
Recurrent mutations are more prevalent in cancer-associated genes. **(A)** The cancer associated exonic splice junction variants recurrence is shown in the pie chart. **(B)** The percentage of tumor suppressor genes, oncogenes and COSMIC cancer gene census (y-axis) genes with the occurrence of variants >=2 and >=5 (x-axis). **(C)** The block bar graph shows the number of unique variants in each gene at the donor and acceptor positions. The top 100 genes with total variant counts and per exon count above 0.4 at each donor or acceptor sites are indicated in the figure. The genes are color-coded and the corresponding variant count per exon for each donor and acceptor position is indicated in the figure

The recurring abundance of variant at the same site in the cancer patients provides compelling evidence for its role in the gene function. We found approximately 17% of the silent and missense variants associated with splice sites occurred at least twice in the COSMIC dataset (Fig. 3A). Disease-associated variants are often linked to specific sets of genes, including tumor suppressors and oncogenes. Mutations in tumor suppressor genes promote uncontrolled cell growth and cancer development, while oncogenes drive tumor formation when mutated or overexpressed. We evaluated the distribution of variants in tumor suppressor genes (TSG), oncogenes, and genes strongly associated with cancer, as listed in the Cancer Gene Census (CGC) curated by COSMIC. Variants with two or more occurrences, approximately 10% are associated with the CGCs, 5% with TSGs, and 4% with oncogenes (Fig. 3B). Among the 758 CGCs, 325 oncogenes, and 324 TSG genes, 530, 224, and 248 genes, respectively, contained at least one variant occurring two or more times (*p* = 3.66E-11, 7.66E-05, 6.81E-12, respectively), whereas 120, 51, and 59 genes, respectively, contained at least one variant occurring five or more times (*p* = 1.48E-29, 7.64E-13, 9.61E-18, respectively). A more detailed examination of variants occurring five or more times unveils that 22% of them (219 sites in 120 genes) are connected to CGC. Among these, 12% are present in TSGs, featuring notable genes such as TP53 (25 variants), VHL (9 variants), and PTEN (7 variants), which are associated with both TSG and CGC (Table S4). Additionally, 12% of the variants are linked to oncogenes, including SF3B1 (6 variants), AR (4 variants), and ALK (2 variants). This analysis underscores the elevated presence of tumor suppressor genes in the splice junction associated missense and silent variants.

An evaluation of frequency variants across all six splice sites per exon in genes revealed an association of key genes with loss-of-function (LoF) as a mechanism of disease development (Fig. 3C, Table S4). Notably, the TP53 tumor suppressor gene was among the top six genes in all donor and acceptor positions in genes with an overall higher frequency of variants (Fig. S3). We observed a high prevalence of CDKN2A variants at donor sites d1 and d2. CDKN2A is a tumor suppressor gene, and its inactivation leads to cancer development. Of the top 5 genes with increased overall variants at the d1 sites, 4 are tumor suppressor genes whose loss of function is the mechanism of disease development. The acceptor site a1 showed a frequency of 1.27 for TP53. The donor site d1 was found to be more frequently associated with CDKN2A, while the a1 site was more commonly linked to TP53. The PTEN gene, characterized by LoF was found in four positions (d1, d2, a1, and a3). We note that some tumor suppressor genes, such as RUNX1, APC, BRAF, MYC, and RB1, are among the most highly enriched genes in the donor and acceptor sites.

**Fig. 4:**
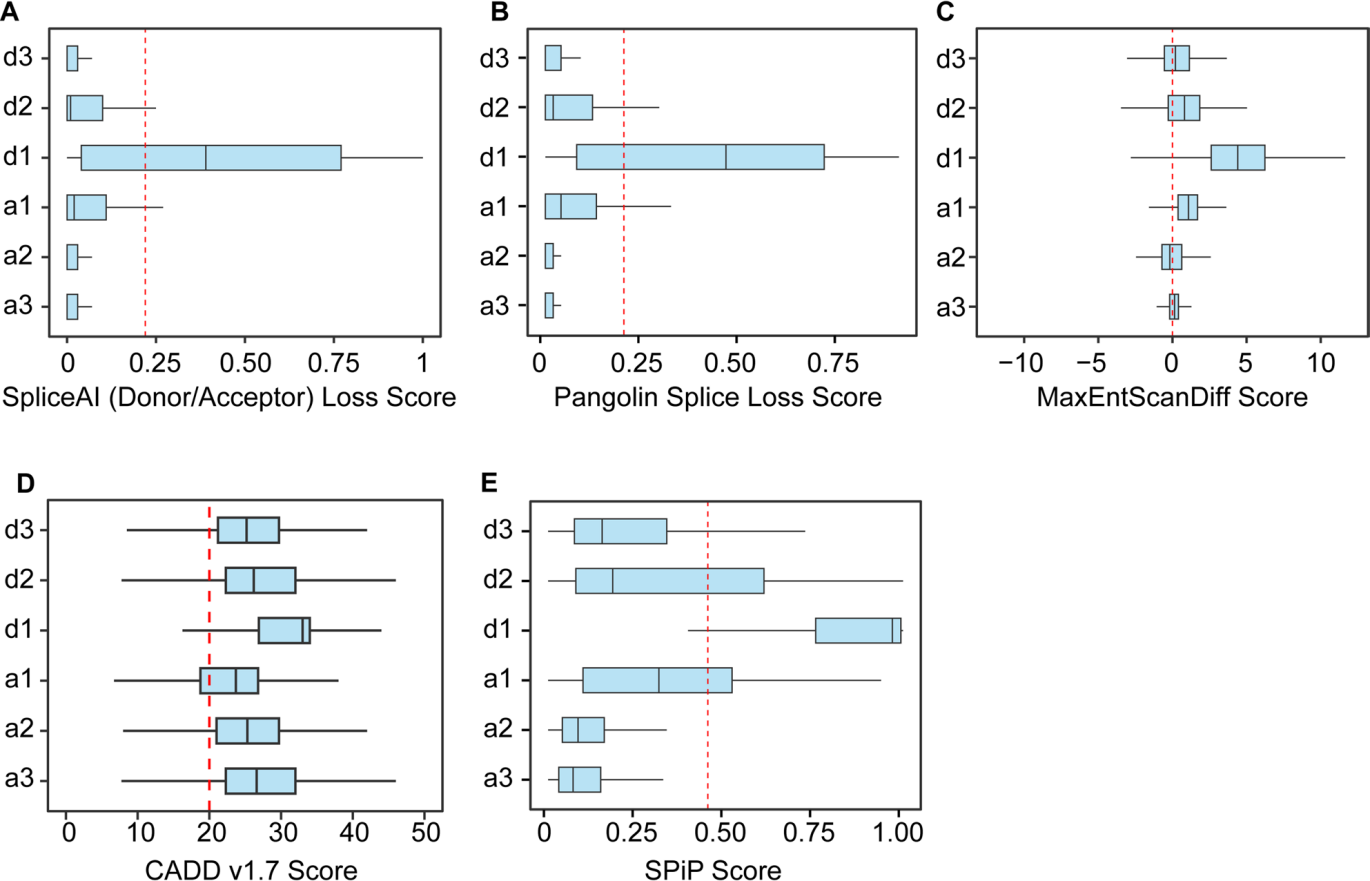
Splice junction variants linked to cancer are predominantly found in loss-of-function genes. **(A)** The top 50 genes with total number of splice junction exonic variants per exon in COSMIC (left), gnomAD (<0.1%) (middle) and gnomAD (>=0.1%) (right). Genes with LOEUF scores <0.33, ≥0.33 and <0.66, ≥0.66 and <1, and ≥1 are color-coded red, orange, yellow, and green, respectively **(B)** Proportion of the top 100 genes across different LOEUF score categories for exonic splice junction variants in COSMIC, gnomAD (<0.1%), and gnomAD (≥0.1%)

The LOEUF (Loss-of-function Observed/Expected Upper Fraction) score is used to assess the degree of constraint on protein-coding genes with respect to loss-of-function (LoF) mutations, such as stop-gain or frameshift mutations. It is a metric developed by the gnomAD (Genome Aggregation Database) consortium using the population data to identify genes that are intolerant to such mutations which can provide insight into the impact of splice site associated silent and missense variants on splicing. We evaluated the association of genes LOEUF scores and their total variant count at all six donor or acceptor sites (Table S5). A total of 56,162 sites (43.65%) were found in genes with LOEUF scores below 0.66. Of these, 82.8% of the sites occur once, while the remaining 17% of the sites occur more than once in cancer patients. Approximately 1% of the sites show at least five occurrences, with 473 sites identified across different cancer types (including stomach, skin, liver, kidney, and lung). A comparative analysis of genes with more frequent occurrences of variants in cancer (COSMIC) and population (gnomAD) datasets revealed a high prevalence of variants in the genes with LOEUF scores below 1 in cancer patients. Interestingly, 29 of the top 50 genes have a LOEUF score of less than 1 (Fig. 4A). These include several key cancer-associated genes with LoF due to truncation or deletion as a mechanism of disease development. However, we observed only a very small number of genes (6 and 8 in MAF < 0.1% and > = 0.1%, respectively) with splice junction exonic variants and a LOEUF score of less than 1 in gnomAD population datasets. Analyzing the top 100 genes in each dataset and comparing their LOEUF scores indicated 46 genes in the COSMIC datasets are characterized by low LOEUF scores (< 0.66), while only 4 genes in the gnomAD dataset (MAF < 0.1%) have LOEUF scores below this threshold (Fig. 4B). Interestingly, only 3 of the top 100 genes in the healthy population (gnomAD < 0.1%) have LOEUF scores below 0.33, while 21 genes in the COSMIC datasets fall below this threshold. A two-sided chi-square test of independence revealed that the differences in proportions of genes with LOEUF scores below 1 across the datasets were statistically significant (*p* < 0.001), indicating that the COSMIC dataset is strongly enriched for genes under constraint relative to gnomAD. This suggests an importance of a proper evaluation of loss-of-function (LoF)-associated mechanism in exonic variants at the splice junction.

### Splice site variants impact prediction tools performance on the exonic variants

**Fig. 5:**
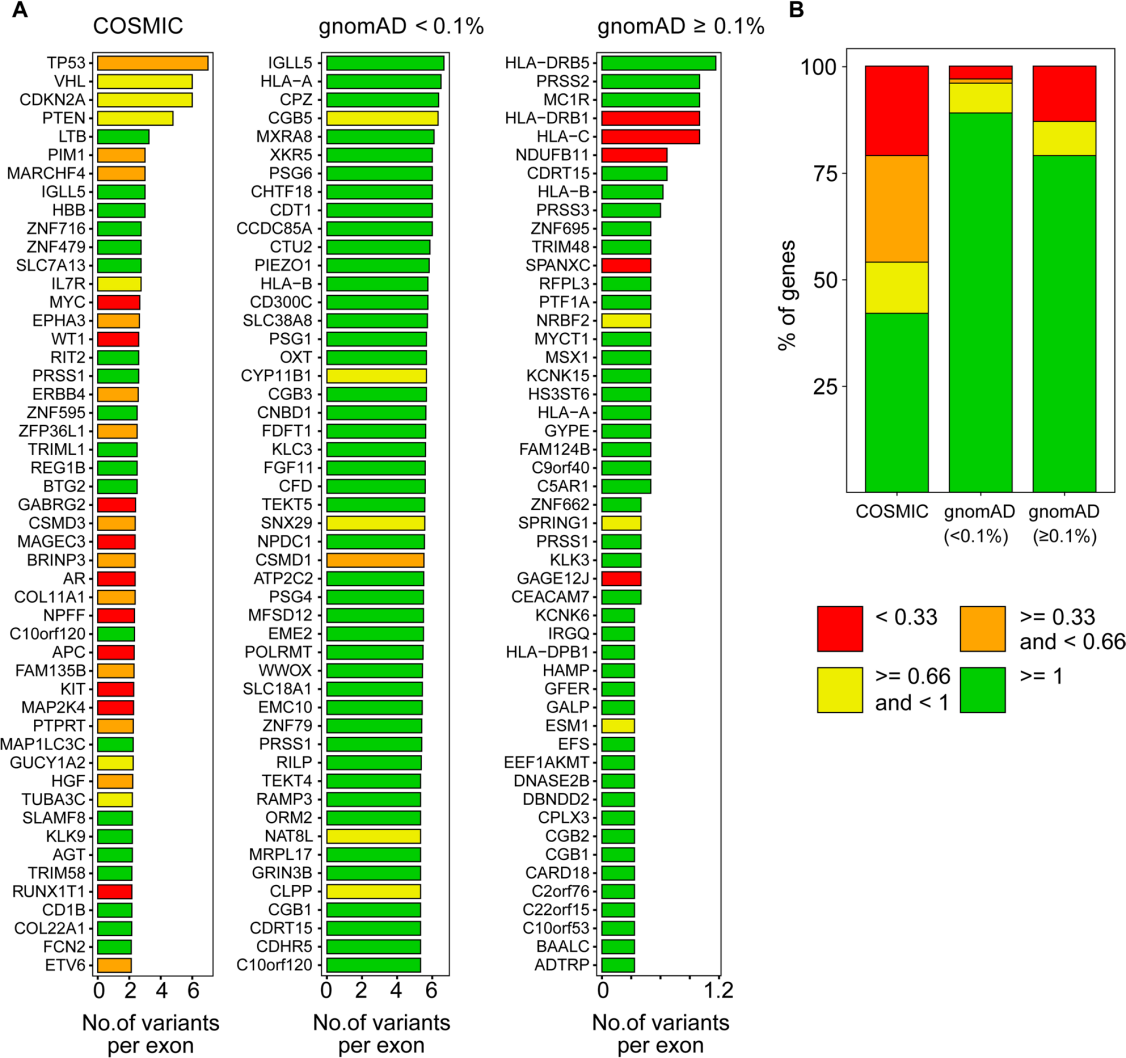
The assessment of silent and missense variants impact on splicing**.** The box plots represent the splice site impact scores predicted by SpliceAI donor/acceptor loss score **(A)**, Pangolin splice loss score **(B)**, MaxEntScan diff score **(C)**, CADD score (v1.7) **(D)**, and SPiP score **(E)** for acceptor (a1-a3) and donor (d1-d3) site variants reported in the COSMIC database. For SpliceAI, the donor sites represent donor loss (DL) and acceptor sites represent acceptor loss (AL) scores. The MaxEntScan diff score represents the difference between the reference and alternate alleles of the donor or acceptor sites

Splice site prediction tools are critical to the prediction of the effect of variants on splicing of the exonic variants. Majority of these tools are designed specifically for donor and acceptor sites and use machine learning, statistical models or neural networks for the prediction. We evaluated the performance of the prediction tools SpliceAI, CADD (v1.7), Pangolin, MaxEntScan and SPiP in predicting the effect of silent and missense variants in the exonic component of the splice junction. We used the variant annotation tools: Variant Effect Predictor (VEP) to derive the prediction score for variants at the donor and acceptor sites. The SpliceAI tool calculates Acceptor Loss (AL), Acceptor Gain (AG), Donor Loss (DL), and Donor Gain (DG) scores for the variants. SpliceAI evaluated 122,863 out of 128,651 sites, with d1 having the highest annotation (*n* = 29360) (Fig. 5A). Variants likely to affect splicing events were identified with a delta score threshold greater than 0.22. The donor loss delta score exceeded 0.22 for the d1 donor site variants, with a median score of 0.39 which indicates most d1 sites variants impact the normal splicing. However, a donor loss score of less than 0.22 was observed in the majority of d2 and d3 sites. The acceptor loss score was comparatively higher at the acceptor sites. However, at the majority of the sites we found the score below 0.22 cut-off. Pangolin is a deep-learning-based method and the prediction scores range from 0 to 1. We obtained the Pangolin score for 128,651 sites. We observed 20,168 donor d1 sites above the 0.20 threshold, indicating that a substantial number of d1 sites have an effect on splicing. Only a small fraction of a1 and d2 sites showed the score above 0.20 (Fig. 5B). MaxEntScan uses a sliding window algorithm (SWA). It evaluates the effect of variants on splicing as determined by reference and alternate allele difference scores for donor or acceptor specific effect. In addition, MaxEntScan also provides a combined difference score. The tool predicted the impact of all the identified variants on splicing. We observed majority of the d1 sites (*n* = 29073) with a median of 4.452 difference score, crossing the minimum threshold. In addition, d2 (*n* = 12826) and d3 (*n* = 11020) also had median donor difference score above 0, indicating that more than half of the variants at these sites are above the threshold (Fig. 5C). Conversely, a substantial proportion of the acceptor sites had an acceptor difference score above the threshold. The median for a1 and a3 positions was above 0. Relative to d1, a small fraction of acceptor sites exhibited a higher splicing loss score. CADD (v1.7) is an advanced prediction model which utilizes conservation scores and several prediction scores to assess the variant impact at the protein and RNA level. It includes splice prediction tools such as MMSplice and SpliceAI for the detailed assessment of potential splice site disruptions. CADD (v1.7) generates splice prediction scores on a scale of 0 to 100, with a suggested threshold cut-off of 20 and above for splice disruption. CADD(v1.7) annotated a total of 99,746 exonic sites in the COSMIC splice junction datasets (Fig. 5D). The donor site d1 and the acceptor site a1 had a median score of 33 and 26.6, respectively. Both sites have a majority of variants above the threshold of 20, indicating a strong likelihood of splice site loss at these positions (Table S6). The sites d2 and d3 have median scores of 26.2 and 25.2, respectively, with more than 64.5% and 78.9% of their annotated sites above the threshold. The Splicing Prediction Pipeline (SPiP) is a machine learning based prediction tool designed for large scale prediction. It provides scores ranging from 0 to 1, with a cutoff of above 0.45 for the splicing impact. The tool annotated all the identified variants and 83% of d1 sites having scores above the cut-off, with a median score of 0.97 (Fig. 5E). In addition, 33%, 17%, and 34% of the d2, d3, and a1 sites, respectively, had prediction scores above the threshold. We evaluated the performance of these tools using experimentally validated splice-impacting variants [45, 46]. A total of 1749 splice-altering variants and 401 non-splice-altering variants were identified.

Receiver operating characteristic (ROC) curve analysis revealed nearly identical area under the curve (AUC) scores of 0.9 for SpliceAI, Pangolin, and MaxEntScan. We observed an AUC score of 0.87 for SPiP and 0.74 for CADD. These scores indicate that splicing-focused prediction tools can perform better in predicting the effect of silent and missense variants at the splice junction (Fig. S4).

### Variants at the splice junctions are highly conserved and functionally important

**Fig. 6:**
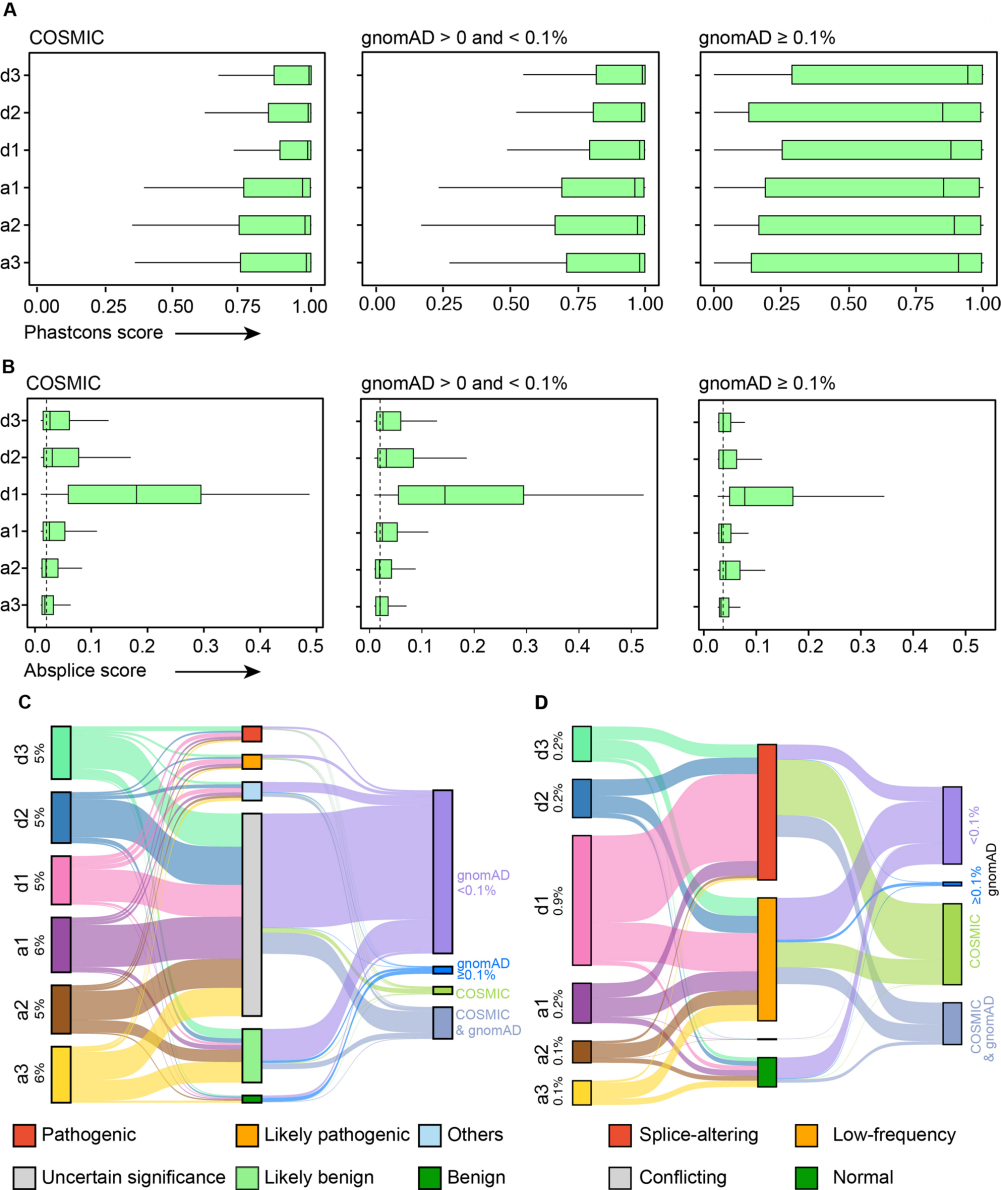
The splice sites associated exonic variants are critical for normal splicing. **(A) **The evolutionary conservation PhastCons 20 score of donor and acceptor sites associated with COSMIC (left), gnomAD (< 0.1%) (middle), and gnomAD (≥ 0.1%) (right) variants. **(B) **The boxplot representing the maximum AbSplice score of donor (d1-d3) and acceptor (a1-a3) sites predicted using 49 GTEx tissues datasets. (**C) **The river plot illustrates the overlap between total human exonic splice junction variants (left), variants reported in the ClinVar database (middle), and those associated with COSMIC and/or gnomAD datasets (right). The total percentage of variants within each group is indicated. River flows are color-coded based on the categories shown on either the left or right side of the plot. ( **D**) The river plot illustrates the overlap of exonic splice junction variants with SpliceVarDB variants, as shown in the panel C

The assessment of the evolutionary conservation is critical because splice sites are preserved by natural selection. The Phastcons score is a nucleotide-level measure of conservation that may indicate a splicing-related role of silent and missense variants at the junction. In the cancer-associated (COSMIC) variants, we observed high Phastcons scores for the majority of sites with median values of 0.99 for positions d1, d2, and d3, 0.98 for a2 and a3, and 0.97 for a1 (Fig. 6A, Table S7). Conversely, in the gnomAD dataset, a broad range of Phastcons scores from 0 to 1 was observed. In the frequent population (gnomAD) variant datasets, representing healthy populations, first-quartile values predominantly around 0.14 to 0.28, with d3 exhibiting the highest median of 0.94. This suggests that a substantial number of variants, particularly those associated with cancer samples, exhibited Phastcons scores close to 1, indicating evolutionary pressure on splice sites.

The impact of exonic splice junction variants is best assessed through transcriptome data, where tools like AbSplice provide critical insight into their potential to disrupt normal splicing. It applies deep learning models trained on large-scale transcriptomic data to assess impact of variant on normal splicing [48]. We evaluated the pre-computed AbSplice score calculated using 49 tissues from Genotype-Tissue Expression (GTEx) dataset (Fig. 6B, Table S8). The AbSplice score between 0.05 and 0.2 indicates a medium likelihood of the aberrant splicing while above 0.2 high likelihood for the aberrant splicing. Of the 25,221 COSMIC donor d1 variants we find 80% of the sites above the 0.05 cut-off while 45% of the sites above 0.2 high likelihood cut-off. In other exonic donor sites we find 31–37% of the variants above 0.05 cut-off (Table S8). The effect of splice site associated silent and missense variants on gene function was assessed by using the impact of the variant on the development of disease as provided in the ClinVar database. It primarily categorizes variants into benign, likely benign, uncertain significance, likely pathogenic and pathogenic based on their clinical significance, disease association, impact on gene function and biological relevance extracted from the literature [38]. We found that 5–6% of donor and acceptor sites were reported in the ClinVar database. A major proportion (65%) of these sites are classified as variants of uncertain significance, which underscores the importance of properly evaluating their impact (Fig. 6C and Table S9). We also searched the ClinVar database for COSMIC and gnomAD variants (Table S9). The COSMIC dataset has a remarkable distribution of variants, with a substantial proportion (2–4%) falling into the pathogenic and likely pathogenic categories (Fig. 6C, Table S9). In position d1, a total of 1584 variants were reported in ClinVar, of which 261 variants (16.4%) were classified as pathogenic/likely-pathogenic, while 103 variants (6.5%) in the benign/likely-benign category. In addition, a major proportion (1020) of the variants in position d1 are classified as uncertain significance, which highlights the need for further validation of these variants. Similarly, in the position a1, a total of 963 variants were found in ClinVar. Of these, 7.2% are classified as pathogenic/likely-pathogenic, 9.4% as benign/likely-benign, and 76% as uncertain significance. In contrast, a relatively higher proportion (ranging from 10% to 22%) of common (gnomAD) variants in the population are classified as benign or likely benign in ClinVar (Fig. 6C). In the a3 position, 314 out of 348 reported variants are benign, while no pathogenic variant has been reported. Similarly, in the a1 position, out of 182 variants reported in ClinVar, 143 variants were classified as benign/likely benign. We also examined the presence of exonic splice variants in SpliceVarDB. This database contains a comprehensive collection of functionally demonstrated splice site variants [45]. We found that 0.1%–0.9% of the variants in exonic splice junctions were reported in SpliceVarDB (Fig. 6D, Table S10). Of these, 0.9% of the d1 site variants were reported in the database. We found that 47% of these variants were reported as splice-altering, while an additional 43% were reported as having a low-frequency impact. Only 10% of the variants were reported as normal, with no impact on splicing. Interestingly, nearly 97% of the reported COSMIC d1 site variants are classified as splice-altering (70%) or low-frequency (27%). Very few sites are reported to have no impact on splicing. This indicates the importance of proper evaluation of variants at exonic splice junctions.

## Discussion

Splicing of pre-mRNA is an essential mechanism to ensure proper removal of introns and in-frame joining of protein-coding exons. Mutations affecting splice sites and splicing regulatory elements can lead to aberrant splicing. The consensus nucleotide motifs present at the exon-intron splice junction are the key components for proper recognition by the spliceosome complex to establish accurate splicing. The nucleotides present on the exonic side of the junction are also important for proper splicing [17]. The exonic component of donor regions contains a [C/A]AG motif in 60% of exons, while the exonic component of acceptor sites contains a G/A motif in 70% of exons. Variants present at these sites are often assessed for their effect on protein function. In a large-scale study of rare natural genetic variants, 3.8% were found to affect splicing; of these, 13% were synonymous and 27% were missense [14]. Several gene-centric screening assays and RNA sequencing data from patient material indicated the effects of missense and silent variants on splicing [45, 54]. A recent study on the coding variants of SPINK1 gene using a full-length gene-splicing assay identified 12 variants that potentially interfere with normal splicing. Of these, 11 are missense variants and 1 is a silent variant. Of these variants, 10 are located within 2 nucleotides of the splice junction. All three possible nucleotide variants (G > N) at donor site 1 of exons 1 and 2, two variants (G > C and G > T) at acceptor site 1 of exon 2, and two variants (A > G and A > T) at d2 of exon 2 could affect normal splicing [55]. A missense variant p.C370Y and a silent variant p.S2479S at the donor site d1 of the VWF gene disrupt the normal splicing and cause exon skipping [33]. Loss of function mutations in the tumor suppressor gene NF1 cause one of the most common diseases, neurofibromatosis type 1. The missense variant p.K1423R in the last three nucleotides of the acceptor arm of exon 32 affects splicing and causes exon skipping [32]. A missense variant, p.Q449H, present at donor site d1 of the TTC8 gene in a patient with retinitis pigmentosa affects splicing and results in premature truncation of the protein [56]. The transcription factor POU1F1 is essential for the normal expression of growth hormone (GH). A high-throughput screening assay of the exon 2 variants identified in patients with pituitary hormone deficiency revealed several splice disruptive silent and missense variants [57]. All three potential variants in the exonic region of donor sites d1 (G > N) and d2 (A > N), as well as only the C > T variant in d3, could affect splicing. At the acceptor site a1 (T > N) and the C > G variant at position a2 also disrupted splicing. These variants could potentially lead to missense or synonymous changes. Mutations in the SCN1A gene are associated with epilepsy phenotypes and functional analysis of 21 reported exonic variants identified 15 variants that affect normal splicing. Four of these variants are located at the d1 donor site, and one is located at the d2 donor site. Additionally, one variant at each acceptor site (a3 and a1) caused splicing impairment [58].

Here, we used somatic variants reported in cancer samples from the COSMIC database to survey the missense and silent variants at splice junctions. We observed 10–17% of the donor and acceptor sites have at least one missense or silent variant. The prevalence of a distinct nucleotide pattern and the enrichment of certain mutation signatures suggest a functional role at the nucleotide level at these positions, rather than at the amino acid level. Several genome-wide studies have focused solely on silent variants to examine their effects on splicing and RNA structure [28–30, 52, 53]. However, in our analysis, we found that 65% to 86% of cancer associated donor and acceptor sites contain missense variants. Available experimental evidence also suggests the impact of several missense variants on RNA splicing (Table S11) [45, 47]. This underscores the need to primarily consider nucleotide-level alterations in the exonic components of the splice junction. The ACMG guidelines recommend up to three nucleotides in the donor site and one nucleotide in the acceptor site of the exons. The current evidences suggest that up to 3 nucleotides in both the directions has to be considered for the experimental and functional evaluations to assess the impact of splicing (Table S11) [45, 47]. In addition, we observed that the vast majority of sites had a high PhastCons score, with a median value greater than 0.95. PhastCons is a measure of evolutionary conservation at the nucleotide level and in a silent (p.E224E) anddicates that these regions are highly conserved. This conservation underscores the functional importance of these regions and reinforces the need for careful assessment of the splicing-associated role of any sequence variation within them.

Tumor suppressor genes and oncogenes frequently harbor driver mutations that are essential for the cancer development. In tumor suppressor genes, loss of function mutation is the most common underlying mechanism that contribute to the disease development. In our analysis, we found that approximately 5% of recurrent mutations (occurrence at least twice) are located in tumor suppressors or oncogenes. In addition, a comprehensive catalogue of cancer associated genes Cancer Gene Census (CGC), curated by COSMIC, are also key genes associated with cancer development. The recurring presence of the same variant across multiple cancer samples serves as an indicator of its potential pathogenicity. We identified about 10% of the variants occurring in at least two samples as part of the CGC genes. In addition, approximately 22% of the sites with at least 5 occurrences are enriched in CGC genes.

Splice-impacting variants at exon boundaries can lead to exon skipping or intron retention, both of which may result in premature protein truncation. The gnomAD consortium derived a metric, LOEUF score, based on the prevalence of protein truncating variants in the healthy population. LOEUF score below 0.66 is considered to be intolerant for the protein function. We identified a total of 56,162 cancer associated sites (44%) located in genes with a LOEUF score below 0.66, of which 17% occur at least twice in the cancer datasets. This highlights the potential relevance of splice-junction variants for tumorigenesis and the necessity to evaluate them for splicing disruption. Protein-truncating variants are typically dispersed throughout the gene locus rather than being concentrated in a specific region of the protein. We found TP53 among the top genes with the highest frequency of variants at donor and acceptor sites, many of which have been experimentally validated. The variants c.G672A and c.G672T, representing a silent (p.E224E) and missense (p.E224D) alteration at the protein level, can induce abnormal splicing that may result in protein truncation [47, 59]. The substitutions at the donor site d1 position 375 of TP53 cDNA (c.G375A, c.G375T, c.G375C), which represents potentially a silent change at the protein level (p.T125T), lead to complete disruption of splicing and cause exon skipping [60, 61]. The variants are prevalent in pediatric adrenocortical tumor which is driven by TP53. However, at position d2 of exon 4 (374), all three nucleotide alterations encoding the missense variants p.T125M, p.T125R, and p.T125K exhibit very mild to no effect on RNA splicing. Interestingly, at the d3 position, the variants c.A373G (p.T125A) and c.A373C (p.T125P) partially affect normal splicing [45, 60]. In addition, plasmids expressing these variants at the d2 and d3 positions are capable of producing full-length proteins, although the transactivation potential of TP53 is reduced as a result of these mutations [60]. This underscores the need for a functional evaluation of the variants impact on splicing. We found a striking association with the genes having a greater number of cancer associated variants with the LOEUF score below 0.66 while very few such genes among top variants from population datasets. This indicates the importance for a detailed evaluation on the impact of exonic variants at the splice junction.

Our evaluation of splice prediction scores indicates that at least 75% of COSMIC variants at site d1 are predicted to affect splicing. For at least 50% of the acceptor site a1 variants, the majority of the tools predict a positive impact on splicing. These predictions were consistent across multiple algorithms, underscoring the utility of integrative approaches in variant interpretation. While evaluating the performance of the tools using experimentally validated variants, we observed nearly equal AUC scores of 0.9 for MaxEntScan, SpliceAI, Pangolin and SPiP, whereas CADD (v1.7) showed a score of 0.74. The relatively lower predictive performance of CADD (v1.7) may be due to the inclusion of several layers of features at the protein, DNA and RNA levels when predicting the impact of the variant. Since our study is restricted to three protein-coding nucleotides in the exonic component of splice junction, the splice centric tools may perform better. Our analysis was also limited by the small number of experimentally validated variants. A large-scale, community-driven experimental evaluation of the identified variants may help to improve the performance and evaluation of these tools. Our comparative analysis of the exonic splice junction variants suggests a higher abundance of variants in skin, lung and endometrial tissues. However, these observations should be interpreted with caution due to the uneven representation of cancer types in COSMIC database and our study focuses on specific exonic regions for characterizing the splicing impact.

A high degree of evolutionary conservation was observed at exonic splice sites associated with cancer datasets, relative to population variants. The PhastCons score, which reflects nucleotide-level conservation, suggests a potential DNA or RNA level functional role for these missense and silent variants at splice junctions. We also evaluated the variants in the ClinVar database for their pathogenicity. The ClinVar database provides a comprehensive collection of variants associated with inherited diseases and cancer. Approximately 5–6% of all exonic donor and acceptor sites are reported in the database. Of these, around 65% are classified as variants of uncertain significance. A large proportion of variants at exonic splice junctions have not been evaluated or are reported as variants of uncertain significance with respect to their impact on gene function and disease development. To address this, a deep learning model such as AbSplice leverage RNA-seq data to predict splice disruption. This approach can help accurately determine the effect of a variant on splicing. Our analysis of the precomputed tissue-specific aberrant splice scores identified that around 45% of COSMIC exonic splice junction variants at the d1 site have a high likelihood of causing aberrant splicing at least in one tissue. A model trained using RNA-seq data from disease cohorts could enable more precise identification of splice-disruptive variants [62]. Our evaluation of experimentally validated splice site exonic variants in the SpliceVarDB indicated that approximately 64% of the reported COSMIC variants are splice-altering, while an additional 34% are categorized as low-frequency with moderate predicted impact. Despite this, only 0.3% of total exonic sites at splice junctions (three nucleotide positions from the donor or acceptor site) have been experimentally evaluated for their impact on splicing.

## Conclusions

Our study underscores the importance of performing comprehensive variant analysis and validation to accurately determine the clinical significance of variants in the intronic and exonic components of splice junctions. Silent and missense variants may demonstrate a neutral effect on protein function; however, evaluating their impact on DNA or RNA function is essential to understand the significance of such variants in disease pathogenesis. Therefore, a thorough, standardized analysis of exonic splice-junction variants is essential for accurate clinical interpretation and precision oncology. Our computational study identified potential exonic splice junction variants associated with splicing in the COSMIC and gnomAD datasets. These datasets lack comprehensive representation of all demographic variables such as age, sex, and ancestry. In addition, the COSMIC database contains uneven distribution of cancer type and certain predicted splice-impacting variants may be enriched or specific to individual tumor types. Furthermore, experimental evaluation of the functional impact of these variants at splice junctions could help stratify splice-impacting variants from those affecting protein function. This would improve the diagnostic process for cancer-associated genes. In particular, variants present in genes for which loss of function is the mechanism of disease development require special attention.

## Supplementary Information

Supplementary Material 1. Fig. S1 The code of splicing junctions

Supplementary Material 2. Fig. S2 The distribution pattern of donor and acceptor sites in cancer tissues

Supplementary Material 3. Fig. S3 The prevalence of splice-altering silent and missense variants at the splice junctions of TP53

Supplementary Material 4. Fig. S4 The splice site prediction tools perform well in identifying the impact of silent and missense variants at the splice junction

Supplementary Material 5. Table S1 Splice junction-associated silent and missense variants observed in the COSMIC and gnomAD databases

Supplementary Material 6. Table S2 Types of nucleotide substitutions associated with silent and missense variants

Supplementary Material 7. Table S3 Summary of exonic splice-junction variant counts across cancer tissue types

Supplementary Material 8. Table S4 Gene-wise total count of variants per exon at donor and acceptor splice sites

Supplementary Material 9. Table S5 Gene-wise summary of total variants per exon at the splice junction

Supplementary Material 10. Table S6 The performance of splice prediction tools in recognizing silent and missense variants

Supplementary Material 11. Table S7 The PhastCons 20-way conservation score of exonic donor and acceptor sites

Supplementary Material 12. Table S8 The AbSplice prediction score of all COSMIC and gnomAD-associated exonic variants in the splice junction

Supplementary Material 13. Table S9 ClinVar assessment of exonic variants at the splice junction

Supplementary Material 14. Table S10 SpliceVarDB assessment of exonic variants at the splice junction

Supplementary Material 15. Table S11 Experimentally validated silent and missense variants affecting normal splicing

## Data Availability

The COSMIC and gnomAD datasets utilized in this study are available from their respective official repositories. All data generated during this study are included in this published article and its supplementary information files.

## Abbreviations

ACMG: American College of Medical Genetics and Genomics
AG: Acceptor Gain
AL: Acceptor Loss
ANNOVAR: ANNOtate VARiation
AUC: Area Under the Curve
CADD: Combined Annotation Dependent Depletion
CGC: Cancer Gene Census
COSMIC: Catalogue Of Somatic Mutations In Cancer
dbNSFP: Database for Nonsynonymous SNPs’ Functional Predictions
DG: Donor Gain
DL: Donor Loss
gnomAD: Genome Aggregation Database
GRCh38: Genome Reference Consortium Human Build 38
GTEx: Genotype-Tissue Expression project
ICGC: International Cancer Genome Consortium
LOEUF: Loss-Of-function Observed/Expected Upper bound Fraction
LoF: Loss of Function
MAF: Minor Allele Frequency
MaxEntScan: Maximum Entropy Scan
PhastCons: Phylogenetic Analysis with Space/Time Models Conservation Scores
ROC: Receiver Operating Characteristic curve
SPiP: Splice Prediction Pipeline
SpliceAI: Splice Artificial Intelligence
TSG: Tumor Suppressor Gene
UTRs: Untranslated Regions
VEP: Variant Effect Predictor
