# Supplementary figures and images for: Synonymous and non-synonymous variants at splice junctions can disrupt splicing and are frequently linked to disease associated loss of function genes
Source: BMC Genomics. 2025 Dec 23;27:99. doi: 10.1186/s12864-025-12466-0 (PMC12838422; doi:10.1186/s12864-025-12466-0)

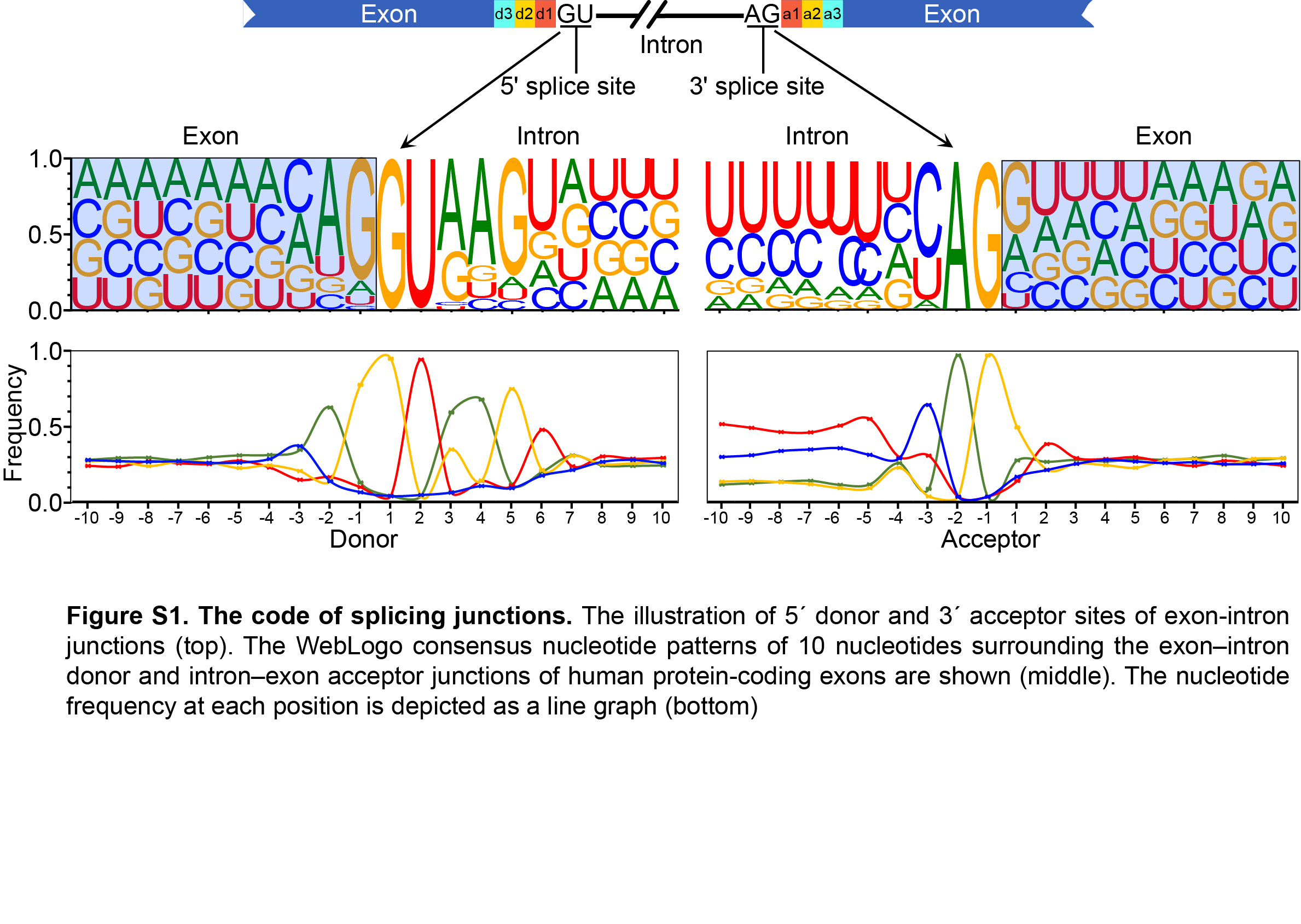

Supplement: Supplementary file 1 — Supplementary Material 1. Fig. S1 The code of splicing junctions [file 12864_2025_12466_MOESM1_ESM.jpg]

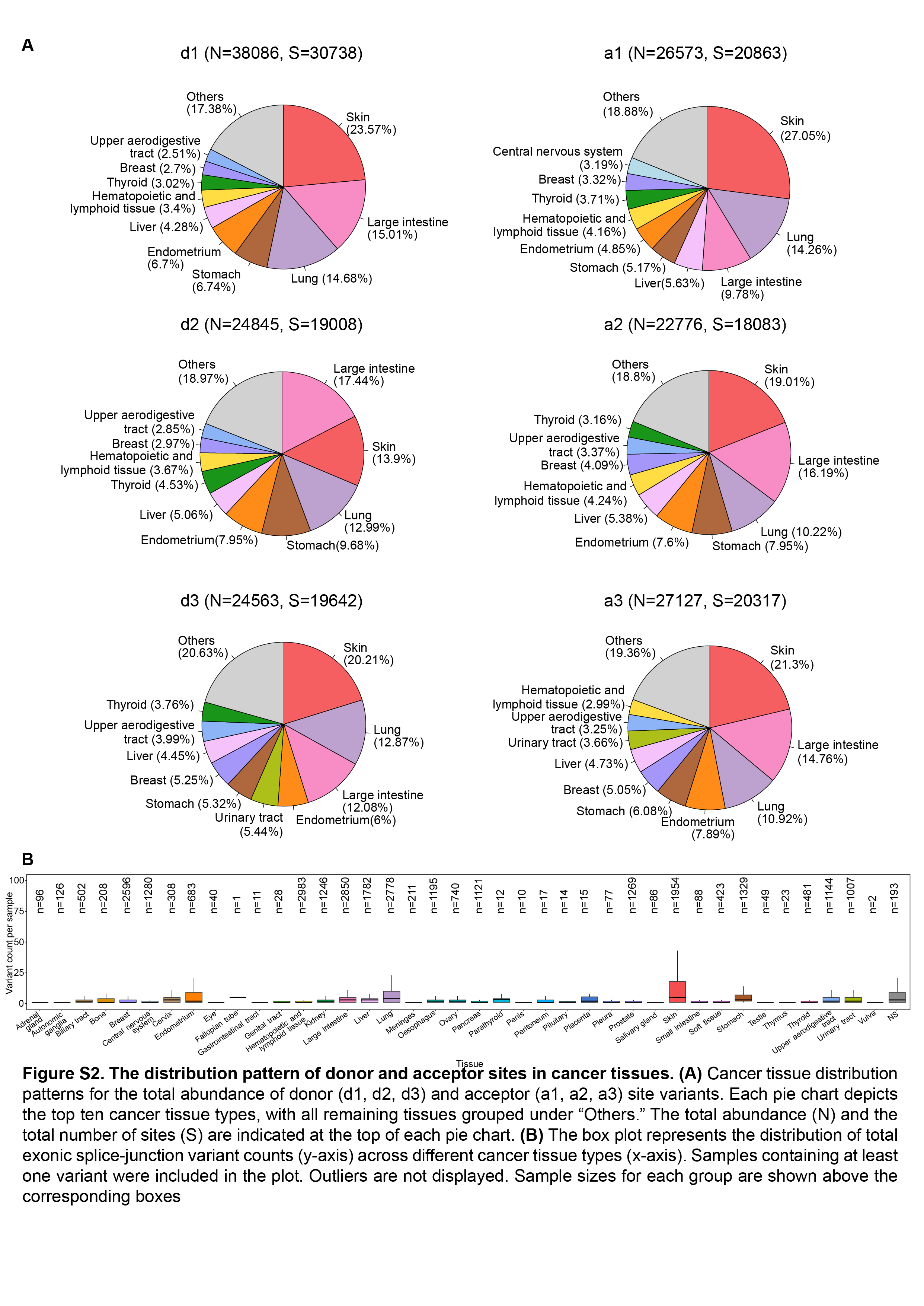

Supplement: Supplementary file 2 — Supplementary Material 2. Fig. S2 The distribution pattern of donor and acceptor sites in cancer tissues [file 12864_2025_12466_MOESM2_ESM.jpg]

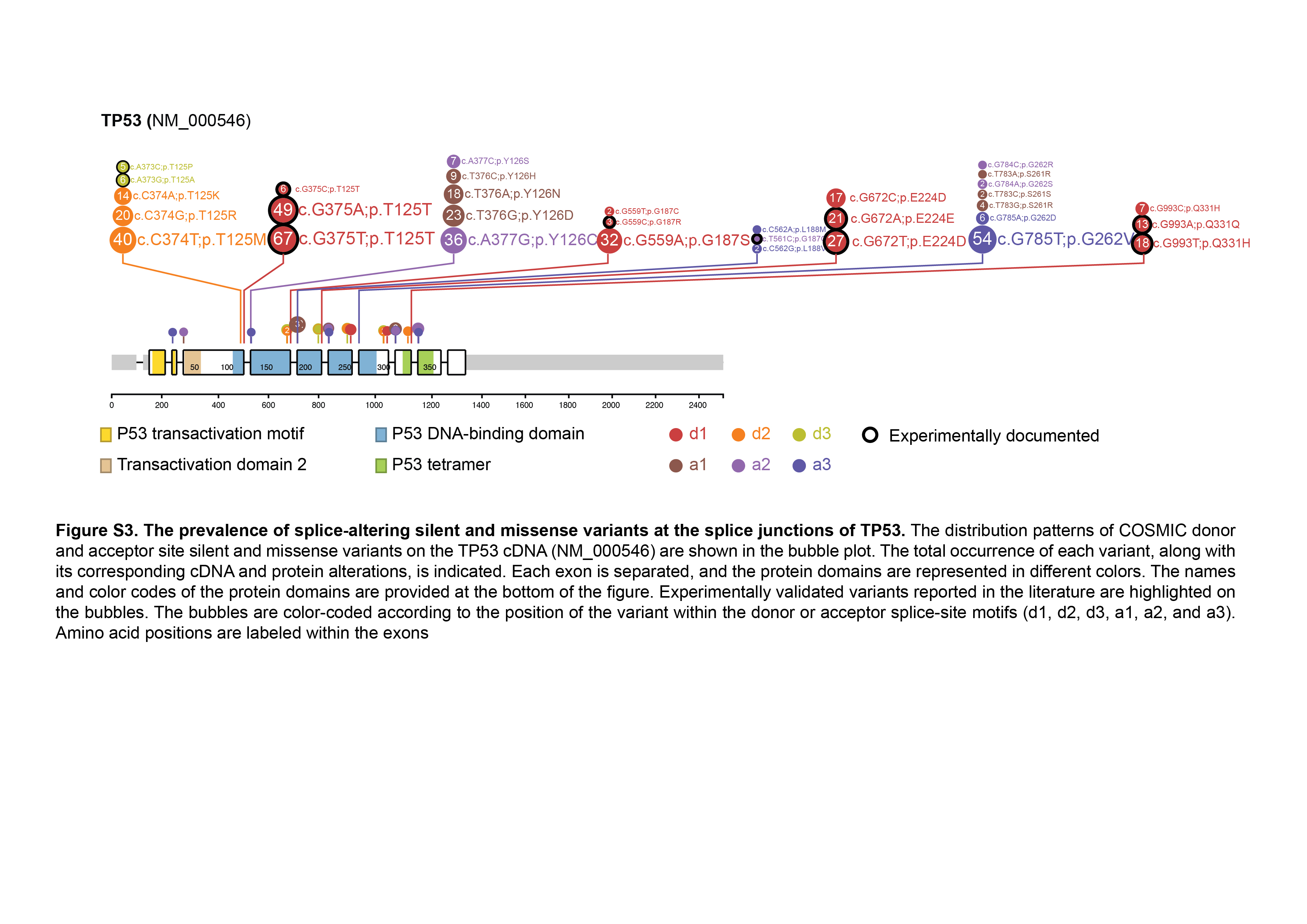

Supplement: Supplementary file 3 — Supplementary Material 3. Fig. S3 The prevalence of splice-altering silent and missense variants at the splice junctions of TP53 [file 12864_2025_12466_MOESM3_ESM.jpg]

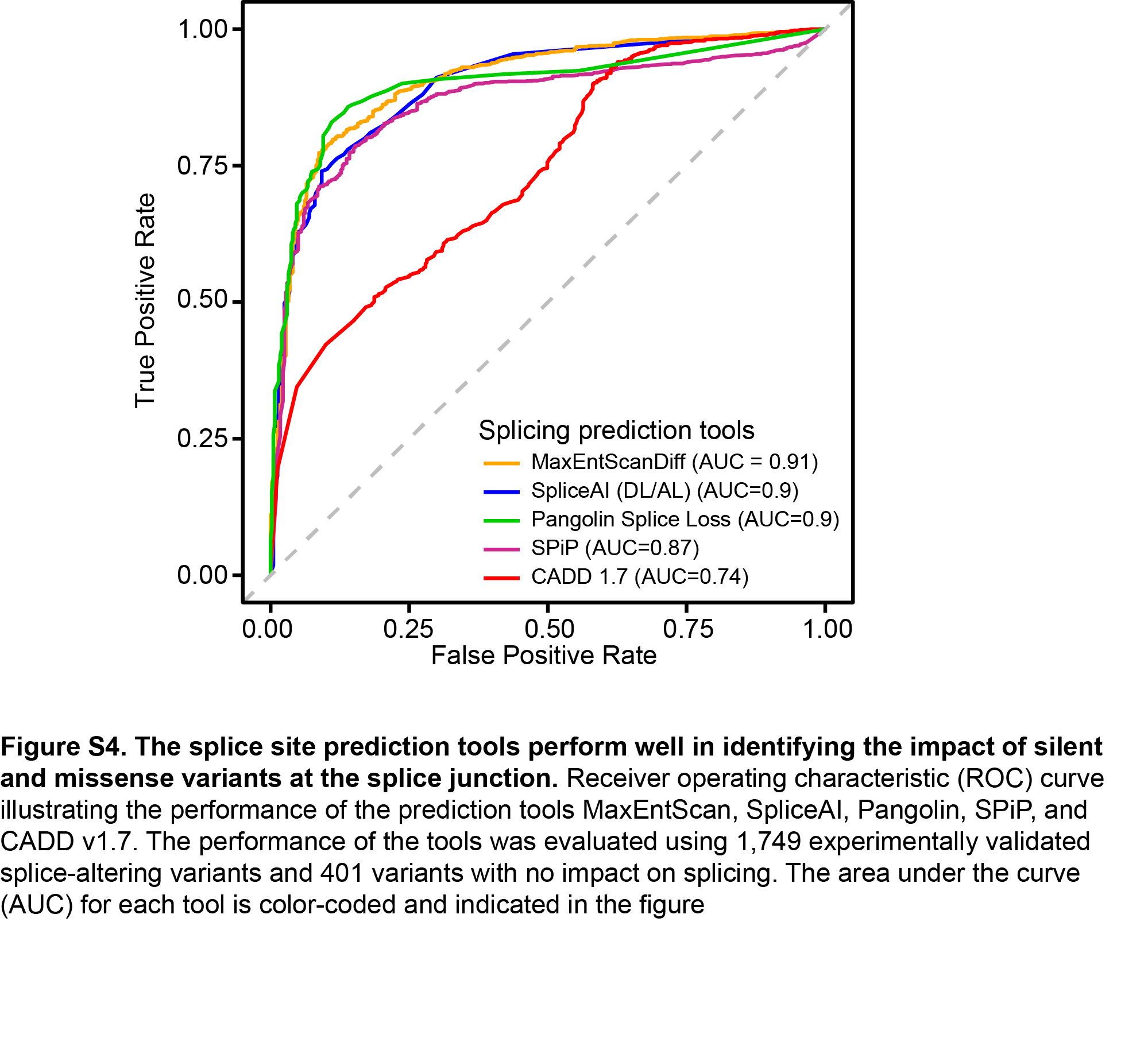

Supplement: Supplementary file 4 — Supplementary Material 4. Fig. S4 The splice site prediction tools perform well in identifying the impact of silent and missense variants at the splice junction [file 12864_2025_12466_MOESM4_ESM.jpg]
