# Supplementary material for: Synonymous and non-synonymous variants at splice junctions can disrupt splicing and are frequently linked to disease associated loss of function genes
Source: BMC Genomics. 2025 Dec 23;27:99. doi: 10.1186/s12864-025-12466-0 (PMC12838422; doi:10.1186/s12864-025-12466-0)
Supplement: Supplementary file 5 — Supplementary Material 5. Table S1 Splice junction-associated silent and missense variants observed in the COSMIC and gnomAD databases [file 12864_2025_12466_MOESM5_ESM.docx]

| **Database** | **Donors** | | | **Acceptors** | | |
| --- | --- | --- | --- | --- | --- | --- |
| **Position** | **d3/-3** | **d2/-2** | **d1/-1** | **a1/+1** | **a2/+2** | **a3/+3** |
| **COSMIC** | Silent:  =(3568/19642)  0.181 (18.1%)  Missense: =(16074/19642)  0.818 (81.8%) | Silent:  =(4813/19008)  0.253 (25.3%)  Missense: =(14195/19008)  0.746 (74.6%) | Silent:  =(6831/30738)  0.222 (22.2%)  Missense: =(23907/30738)  0.777 (77.7%) | Silent:  =(2900/20863)  0.139 (13.9%)  Missense: =(17963/20863)  0.861 (86.1%) | Silent:  =(4838/18083)  0.26.7 (26.7%)  Missense: =(13245/18083)  0.732 (73.2%) | Silent:  =(6977/20317)  0.343 (34.3%)  Missense: =(13340/20317)  0.656 (65.6%) |
| **gnomAD (>0 and <0.1%)** | Silent:  =(18644/83147)  0.224 (22.4%)  Missense: =(64503/83147)  0.775 (77.5%) | Silent:  =(27203/87018)  0.312 (31.2%)  Missense: =(59815/87018)  0.687 (68.7%) | Silent:  =(31770/100113)  0.317 (31.7%)  Missense: =(68343/100113)  0.682 (68.2%) | Silent:  =(16058/98217)  0.163 (16.3%)  Missense: =(82159/98217)  0.836 (83.6%) | Silent:  =(26826/84573)  0.317 (31.7%)  Missense: =(57747/84573)  0.682 (68.2%) | Silent:  =(38339/94182)  0.407 (40.7%)  Missense: =(55843/94182)  0.592 (59.2s%) |
| **gnomAD (>=0.1%)** | Silent:  =(183/542)  0.337 (33.7%)  Missense: =(359/542)  0.662 (66.2%) | Silent:  =(291/671)  0.433 (43.3%)  Missense: =(380/671)  0.566 (56.6%) | Silent:  =(261/482)  0.541 (54.1%)  Missense: =(221/482)  0.458 (45.8%) | Silent:  =(136/471)  0.288 (28.8%)  Missense: =(335/471)  0.711 (71.1%) | Silent:  =(266/580)  0.458 (45.8%)  Missense: =(314/580)  0.541 (54.1%) | Silent:  =(435/778)  0.559 (55.9%)  Missense: =(343/778)  0.440 (44%) |

**Table S1. Splice junction-associated silent and missense variants observed in the COSMIC and gnomAD databases**

The count and percentage of silent and missense variants observed in the last three nucleotide positions of donor and acceptor splice sites across three datasets: COSMIC, gnomAD >0 and <0.1%, and gnomAD ≥0.1%. Rows display the count of silent and missense variants for each dataset, while the columns represent the six splice sites of donor and acceptor regions, respectively.
