# Supplementary material for: Synonymous and non-synonymous variants at splice junctions can disrupt splicing and are frequently linked to disease associated loss of function genes
Source: BMC Genomics. 2025 Dec 23;27:99. doi: 10.1186/s12864-025-12466-0 (PMC12838422; doi:10.1186/s12864-025-12466-0)
Supplement: Supplementary file 6 — Supplementary Material 6. Table S2 Types of nucleotide substitutions associated with silent and missense variants [file 12864_2025_12466_MOESM6_ESM.docx]

**Table S2. Types of nucleotide substitutions associated with silent and missense variants**

**COSMIC**

| **Variant** | **Silent** | | | | | | **Missense** | | | | | |
| --- | --- | --- | --- | --- | --- | --- | --- | --- | --- | --- | --- | --- |
| **Position** | **d3** | **d2** | **d1** | **a1** | **a2** | **a3** | **d3** | **d2** | **d1** | **a1** | **a2** | **a3** |
| **A>C** | 59 | 331 | 50 | 18 | 60 | 76 | 579 | 1115 | 348 | 302 | 348 | 441 |
| **A>G** | 218 | 872 | 150 | 177 | 357 | 440 | 1555 | 2863 | 404 | 1003 | 1071 | 1151 |
| **A>T** | 49 | 375 | 45 | 29 | 112 | 142 | 464 | 1740 | 266 | 599 | 484 | 549 |
| **C>A** | 498 | 285 | 85 | 49 | 315 | 691 | 2372 | 749 | 299 | 804 | 975 | 1275 |
| **C>G** | 147 | 43 | 7 | 11 | 107 | 195 | 986 | 267 | 117 | 323 | 349 | 611 |
| **C>T** | 1543 | 1505 | 668 | 616 | 1130 | 2437 | 2954 | 2933 | 1265 | 1410 | 2241 | 2611 |
| **G>A** | 616 | 564 | 4578 | 1492 | 1276 | 1389 | 3780 | 1512 | 7493 | 7011 | 2803 | 2953 |
| **G>C** | 64 | 62 | 156 | 61 | 69 | 105 | 1353 | 462 | 2058 | 1247 | 407 | 569 |
| **G>T** | 118 | 347 | 894 | 232 | 354 | 529 | 1182 | 1644 | 11282 | 4286 | 1532 | 1658 |
| **T>A** | 25 | 64 | 37 | 12 | 121 | 129 | 249 | 198 | 124 | 218 | 631 | 317 |
| **T>C** | 205 | 320 | 136 | 192 | 724 | 674 | 399 | 502 | 162 | 516 | 1570 | 634 |
| **T>G** | 26 | 45 | 25 | 11 | 213 | 170 | 201 | 210 | 89 | 244 | 834 | 571 |

**gnomAD >=0.1%**

| **Variant** | **Silent** | | | | | | **Missense** | | | | | |
| --- | --- | --- | --- | --- | --- | --- | --- | --- | --- | --- | --- | --- |
| **Position** | **d3** | **d2** | **d1** | **a1** | **a2** | **a3** | **d3** | **d2** | **d1** | **a1** | **a2** | **a3** |
| **A>C** | 5 | 9 | 5 | 2 | 8 | 16 | 25 | 17 | 3 | 15 | 30 | 18 |
| **A>G** | 26 | 36 | 27 | 8 | 26 | 55 | 47 | 42 | 14 | 32 | 36 | 27 |
| **A>T** | 1 | 3 | 1 | 1 | 7 | 6 | 6 | 17 | 4 | 9 | 6 | 14 |
| **C>A** | 7 | 4 | 3 | 1 | 6 | 9 | 23 | 10 | 13 | 22 | 18 | 20 |
| **C>G** | 9 | 5 | 4 | 3 | 4 | 15 | 21 | 14 | 19 | 26 | 16 | 13 |
| **C>T** | 41 | 81 | 103 | 45 | 87 | 118 | 69 | 77 | 61 | 72 | 49 | 75 |
| **G>A** | 49 | 86 | 88 | 39 | 69 | 114 | 67 | 88 | 51 | 71 | 61 | 61 |
| **G>C** | 6 | 10 | 2 | 2 | 3 | 13 | 25 | 16 | 24 | 22 | 15 | 21 |
| **G>T** | 7 | 14 | 1 | 3 | 13 | 10 | 10 | 11 | 8 | 20 | 15 | 26 |
| **T>A** | 4 | 5 | 0 | 1 | 4 | 8 | 2 | 22 | 3 | 7 | 12 | 11 |
| **T>C** | 23 | 31 | 27 | 30 | 26 | 58 | 47 | 50 | 17 | 26 | 32 | 43 |
| **T>G** | 5 | 7 | 0 | 1 | 13 | 13 | 17 | 16 | 4 | 13 | 24 | 14 |

**gnomAD <0.1%**

| **Variant** | **Silent** | | | | | | **Missense** | | | | | |
| --- | --- | --- | --- | --- | --- | --- | --- | --- | --- | --- | --- | --- |
| **Position** | **d3** | **d2** | **d1** | **a1** | **a2** | **a3** | **d3** | **d2** | **d1** | **a1** | **a2** | **a3** |
| **A>C** | 504 | 1820 | 284 | 197 | 904 | 993 | 3165 | 4360 | 1294 | 2300 | 3178 | 2729 |
| **A>G** | 2308 | 5723 | 2131 | 1856 | 3812 | 5281 | 7981 | 11229 | 2649 | 6479 | 7352 | 6171 |
| **A>T** | 209 | 919 | 204 | 142 | 864 | 993 | 1606 | 3575 | 865 | 2605 | 3094 | 2388 |
| **C>A** | 1002 | 903 | 1102 | 572 | 1755 | 2306 | 4652 | 2288 | 7547 | 9029 | 4457 | 4503 |
| **C>G** | 761 | 651 | 920 | 379 | 990 | 1488 | 6146 | 2655 | 8770 | 6269 | 3091 | 3859 |
| **C>T** | 4603 | 3583 | 11218 | 4876 | 5096 | 8047 | 8685 | 5615 | 13296 | 14462 | 7746 | 7867 |
| **G>A** | 4643 | 3481 | 11244 | 4864 | 5210 | 8051 | 8911 | 5775 | 13487 | 14572 | 7880 | 8185 |
| **G>C** | 774 | 602 | 913 | 375 | 954 | 1424 | 6172 | 2747 | 8742 | 6348 | 3252 | 4034 |
| **G>T** | 996 | 848 | 1073 | 589 | 1613 | 2235 | 4368 | 2224 | 6958 | 8722 | 4311 | 4470 |
| **T>A** | 206 | 955 | 186 | 125 | 843 | 1042 | 1622 | 3584 | 802 | 2661 | 3088 | 2459 |
| **T>C** | 2158 | 5864 | 2160 | 1906 | 3797 | 5410 | 7968 | 11292 | 2694 | 6362 | 7276 | 6464 |
| **T>G** | 480 | 1854 | 335 | 177 | 988 | 1069 | 3227 | 4471 | 1239 | 2350 | 3022 | 2714 |

The nucleotide substitutions for silent and missense variants occur at the last three nucleotide positions of acceptor and donor splice sites. The data are categorized into three groups: COSMIC, gnomAD >0 and <0.1%, and gnomAD ≥0.1%. Rows indicate the nucleotide substitutions for all the variants, while the columns represent the six splice site positions of donor and acceptor regions.
