# Supplementary material for: Synonymous and non-synonymous variants at splice junctions can disrupt splicing and are frequently linked to disease associated loss of function genes
Source: BMC Genomics. 2025 Dec 23;27:99. doi: 10.1186/s12864-025-12466-0 (PMC12838422; doi:10.1186/s12864-025-12466-0)
Supplement: Supplementary file 7 — Supplementary Material 7. Table S3 Summary of exonic splice-junction variant counts across cancer tissue types [file 12864_2025_12466_MOESM7_ESM.docx]

**Table S3. Summary of exonic splice-junction variant counts across cancer tissue types**

| Cancer tissue type | Total Samples | Mean Variant Count | Median (Range) |
| --- | --- | --- | --- |
| Adrenal gland | 2573 | 0.063 | 0 (0, 16) |
| Autonomic ganglia | 1303 | 0.135 | 0 (0, 8) |
| Biliary tract | 3551 | 0.605 | 0 (0, 76) |
| Bone | 2422 | 0.237 | 0 (0, 12) |
| Breast | 17767 | 0.35 | 0 (0, 128) |
| Central nervous system | 17572 | 0.246 | 0 (0, 331) |
| Cervix | 1227 | 1.217 | 0 (0, 50) |
| Endometrium | 5147 | 2.154 | 0 (0, 427) |
| Eye | 1700 | 0.025 | 0 (0, 3) |
| Fallopian tube | 13 | 0.385 | 0 (0, 5) |
| Female genital tract | 55 | 0 | 0 (0, 0) |
| Female genitourinary system | 17 | 0 | 0 (0, 0) |
| Gastrointestinal tract | 209 | 0.057 | 0 (0, 2) |
| Genital tract | 274 | 0.274 | 0 (0, 27) |
| Hematopoietic and lymphoid tissue | 125603 | 0.047 | 0 (0, 71) |
| Kidney | 6913 | 0.453 | 0 (0, 57) |
| Large intestine | 55354 | 0.42 | 0 (0, 281) |
| Liver | 5419 | 1.495 | 0 (0, 242) |
| Lung | 45280 | 0.463 | 0 (0, 112) |
| Mediastinum | 1 | 0 | 0 (0, 0) |
| Meninges | 1799 | 0.163 | 0 (0, 13) |
| Oesophagus | 4369 | 0.745 | 0 (0, 56) |
| Ovary | 6656 | 0.313 | 0 (0, 167) |
| Pancreas | 10506 | 0.239 | 0 (0, 49) |
| Paratesticular tissues | 5 | 0 | 0 (0, 0) |
| Parathyroid | 344 | 0.116 | 0 (0, 8) |
| Penis | 179 | 0.123 | 0 (0, 12) |
| Pericardium | 2 | 0 | 0 (0, 0) |
| Perineum | 1 | 0 | 0 (0, 0) |
| Peritoneum | 310 | 0.21 | 0 (0, 27) |
| Pituitary | 1079 | 0.02 | 0 (0, 4) |
| Placenta | 39 | 9.026 | 0 (0, 298) |
| Pleura | 883 | 0.129 | 0 (0, 8) |
| Prostate | 5354 | 0.564 | 0 (0, 206) |
| Retroperitoneum | 1 | 0 | 0 (0, 0) |
| Salivary gland | 900 | 0.213 | 0 (0, 31) |
| Skin | 20712 | 1.668 | 0 (0, 548) |
| Small intestine | 941 | 0.456 | 0 (0, 153) |
| Soft tissue | 14741 | 0.072 | 0 (0, 71) |
| Stomach | 5140 | 2.171 | 0 (0, 683) |
| Testis | 577 | 0.123 | 0 (0, 6) |
| Thymus | 204 | 0.157 | 0 (0, 4) |
| Thyroid | 30426 | 0.185 | 0 (0, 135) |
| Upper aerodigestive tract | 6105 | 0.835 | 0 (0, 75) |
| Urinary tract | 7391 | 0.604 | 0 (0, 88) |
| Uterine adnexa | 14 | 0 | 0 (0, 0) |
| Vagina | 8 | 0 | 0 (0, 0) |
| Vulva | 210 | 0.01 | 0 (0, 1) |
| NS | 3693 | 0.505 | 0 (0, 364) |

Kruskal-Wallis test (2.2E-16)

The total number of samples analyzed, the mean variant count, and the median variant count with range are shown for each cancer tissue type.
