# Supplementary material for: Synonymous and non-synonymous variants at splice junctions can disrupt splicing and are frequently linked to disease associated loss of function genes
Source: BMC Genomics. 2025 Dec 23;27:99. doi: 10.1186/s12864-025-12466-0 (PMC12838422; doi:10.1186/s12864-025-12466-0)
Supplement: Supplementary file 10 — Supplementary Material 10. Table S6 The performance of splice prediction tools in recognizing silent and missense variants [file 12864_2025_12466_MOESM10_ESM.docx]

**Table S6. The performance of splice prediction tools in recognizing silent and missense variants**

| **Site** | **SpliceAI_**  **Acceptor loss (>0.22)** | **SpliceAI_**  **Donor loss (>0.22)** | **Pangolin Splice Loss(>0.2)** | **CADD(>20)** | **MAX_SWA Acceptor_diff (>0)** | **MAX_SWA Donor_diff (>0)** | **MaxEntScan_diff (>0)** | **SPiP (>0.45)** |
| --- | --- | --- | --- | --- | --- | --- | --- | --- |
| **d3** | Total: 48/18702,  Min: 0,  Q1: 0, Median: 0, Q3: 0, Max: 0.77 | Total: 1104/18702,  Min: 0,  Q1: 0, Median: 0, Q3: 0.03,  Max: 0.97 | Total: 1476/19642,  Min: -0.9,  Q1: -0.04, Median: 0, Q3: 0, Max: 0 | Total: 12741/16155,  Min: 0.001, Q1: 21.2, Median: 25.2, Q3: 29.7,  Max: 60 | Total: 7923/19642,  Min: -19.976, Q1: -3.401, Median: -0.286, Q3: 0.477,  Max: 13.653 | Total: 10724/19642,  Min: -13.042, Q1: -0.59, Median: 0.113, Q3: 1.072,  Max: 8.182 | Total: 11020/19633,  Min: -4.062,  Q1: -0.544,  Median: 0.197,  Q3: 1.139,  Max: 5.337 | Total: 3408/19642,  Min: -1,  Q1: 0.074, Median: 0.152, Q3: 0.334,  Max: 0.996 |
| **d2** | Total: 130/18173,  Min: 0,  Q1: 0, Median: 0, Q3: 0, Max: 0.85 | Total: 2882/18173,  Min: 0,  Q1: 0, Median: 0.01, Q3: 0.1,  Max: 0.99 | Total: 3680/19008,  Min: -0.85,  Q1: -0.12, Median: -0.02, Q3: 0, Max: 0 | Total: 11706/14314,  Min: 0.001, Q1: 22.3, Median: 26.2, Q3: 32,  Max:50 | Total: 5489/19008,  Min: -27.765, Q1: -4.8518, Median: -0.935, Q3: 0.1643,  Max: 8.750 | Total: 12739/19008,  Min: -11.863, Q1: -0.318, Median: 0.8, Q3: 1.826,  Max: 8.182 | Total: 12826/19005,  Min: -6.854,  Q1: -0.286,  Median: 0.807,  Q3: 1.838,  Max: 8.759 | Total: 6200/19008,  Min: -1,  Q1: 0.078, Median:0.182,  Q3: 0.608,  Max: 1 |
| **d1** | Total: 819/29360,  Min: 0,  Q1: 0, Median: 0, Q3: 0,  Max: 0.97 | Total: 17381/29360,  Min: 0,  Q1: 0.04, Median: 0.39, Q3: 0.77,  Max: 1 | Total: 20168/30738,  Min: -0.9,  Q1: -0.71, Median: -0.46, Q3: -0.08, Max: 0 | Total: 22629/24038,  Min: 0.001, Q1: 26.9, Median: 33, Q3: 34,  Max: 79 | Total: 5846/30738,  Min: -15.709, Q1: -7.576, Median: -2.362, Q3: -0.459,  Max: 8.75 | Total: 27555/30738,  Min: -14.484, Q1: 2.217, Median: 4.098, Q3: 5.821,  Max: 15.796 | Total: 29073/30726,  Min: -11.522,  Q1: 2.638,  Median: 4.452,  Q3: 6.385,  Max: 20.926 | Total: 25397/30738,  Min: -1,  Q1: 0.752, Median: 0.97, Q3: 0.994,  Max: 1 |
| **a1** | Total: 2716/19946,  Min: 0,  Q1: 0, Median: 0.02, Q3: 0.11, Max: 1 | Total: 142/19946,  Min: 0,  Q1: 0, Median: 0, Q3: 0,  Max: 0.89 | Total: 3731/20863,  Min: -0.78,  Q1: -0.13, Median: -0.04, Q3: 0, Max: 0 | Total: 14978/18142,  Min: 0.001, Q1: 22.3, Median: 26.6, Q3: 32,  Max: 57 | Total: 16660/20863,  Min: -26.087, Q1: 0.3105, Median: 1.054, Q3: 1.673,  Max: 5.476 | Total: 10472/20863,  Min: -16.34, Q1: -4.988, Median: 0.04, Q3: 8.182,  Max:14.16 | Total: 16901/20855,  Min: -20.859,  Q1: 0.378,  Median: 1.081,  Q3: 1.687,  Max: 6.688 | Total: 7005/20863,  Min: -1,  Q1: 0.098, Median: 0.31, Q3: 0.518,  Max: 0.938 |
| **a2** | Total: 697/17222,  Min: 0,  Q1: 0, Median: 0, Q3: 0.03, Max: 1 | Total: 16/17222,  Min: 0,  Q1: 0, Median: 0, Q3: 0,  Max: 0.51 | Total: 416/18083,  Min: -0.79,  Q1: -0.02, Median: 0, Q3: 0, Max: 0 | Total: 10512/13503,  Min: 0.001, Q1: 21, Median: 25.3, Q3: 29.7,  Max: 52 | Total: 7736/18083,  Min: -8.75, Q1: -0.714, Median: -0.185, Q3: 0.599,  Max: 6.097 | Total: 6358/18083,  Min: -20.343, Q1: -7.754, Median: -2.173, Q3: 0.536,  Max: 12.428 | Total: 7789/18077,  Min: -9.008,  Q1: -0.708,  Median: -0.178,  Q3: 0.621,  Max: 7.955 | Total: 399/18083,  Min: -1,  Q1: 0.04, Median: 0.084, Q3: 0.158,  Max: 0.93 |
| **a3** | Total: 896/19460,  Min: 0,  Q1: 0, Median: 0, Q3: 0.03, Max: 1 | Total: 25/19460,  Min: 0,  Q1: 0, Median: 0, Q3: 0,  Max: 0.61 | Total: 253/20317,  Min: -0.76,  Q1: -0.02, Median: 0, Q3: 0, Max: 0 | Total: 9667/13594,  Min: 0.001, Q1: 18.76, Median: 23.7, Q3: 26.8,  Max: 53 | Total: 12347/20317,  Min: -8.75, Q1: -0.203, Median: 0.14, Q3: 0.399,  Max: 2.167 | Total: 7077/20317,  Min: -20.942, Q1: -4.822, Median: -1.547, Q3: 0.981,  Max: 12.959 | Total: 12424/20309,  Min: -2.508,  Q1: -0.189,  Median: 0.14,  Q3: 0.399,  Max: 8.75 | Total: 558/20317,  Min: -1,  Q1: 0.03, Median: 0.07, Q3: 0.148,  Max: 0.944 |

The performance of splicing prediction tools on COSMIC silent and missense splice junction variants is summarized. Rows represent the six splice site positions of both donor and acceptor sites, while the columns correspond to six different splicing prediction tools: SpliceAI, Pangolin, CADD v1.7, MaxEntScan, and SPiP. Each cell provides a detailed statistical summary of the predicted splicing positions for the respective tool and splice site position. This summary includes the number of variants above the threshold (blue) and the total count of positions annotated by the tool, along with five key statistical measures: minimum (min), first quartile (Q1), median (med), third quartile (Q3), and maximum (max). The recommended minimum score of each tool for the positive prediction of splice sites is indicated in the column names (red). The MaxEntScan donor, acceptor, and combined difference scores, and SpliceAI acceptor loss and donor loss scores are provided in different columns
