# Supplementary material for: Synonymous and non-synonymous variants at splice junctions can disrupt splicing and are frequently linked to disease associated loss of function genes
Source: BMC Genomics. 2025 Dec 23;27:99. doi: 10.1186/s12864-025-12466-0 (PMC12838422; doi:10.1186/s12864-025-12466-0)
Supplement: Supplementary file 11 — Supplementary Material 11. Table S7 The PhastCons 20-way conservation score of exonic donor and acceptor sites [file 12864_2025_12466_MOESM11_ESM.docx]

**Table S7. The PhastCons 20-way conservation score of exonic donor and acceptor sites**

| **Position** | **COSMIC** | **gnomAD**  **(>0 and <0.1%)** | **gnomAD**  **(>=0.1%)** |
| --- | --- | --- | --- |
| **D3** | Total: 19641,  Min: 0,  Q1: 0.861,  Median: 0.991,  Q3: 0.999,  Max: 1 | Total: 83147,  Min: 0,  Q1: 0.818,  Median: 0.989,  Q3: 0.999,  Max: 1 | Total: 542,  Min: 0,  Q1: 0.2892,  Median: 0.942,  Q3: 0.996,  Max: 1 |
| **D2** | Total: 19007,  Min: 0,  Q1: 0.84,  Median: 0.988,  Q3: 0.998,  Max: 1 | Total: 87018,  Min: 0,  Q1: 0.807,  Median: 0.986,  Q3: 0.998,  Max: 1 | Total: 671,  Min: 0,  Q1: 0.129,  Median: 0.849,  Q3: 0.99,  Max: 1 |
| **D1** | Total: 30735,  Min: 0,  Q1: 0.883,  Median: 0.986,  Q3: 0.998,  Max: 1 | Total: 100113,  Min: 0,  Q1: 0.793,  Median: 0.979,  Q3: 0.997,  Max: 1 | Total: 482,  Min: 0,  Q1: 0.253,  Median: 0.8795,  Q3: 0.993,  Max: 1 |
| **A1** | Total: 20863,  Min: 0,  Q1: 0.748,  Median: 0.967,  Q3: 0.996,  Max: 1 | Total: 98217,  Min: 0,  Q1: 0.69,  Median: 0.961,  Q3: 0.995,  Max: 1 | Total: 471,  Min: 0,  Q1: 0.1905,  Median: 0.852,  Q3: 0.9845,  Max: 1 |
| **A2** | Total: 18083,  Min: 0,  Q1: 0.731,  Median: 0.976,  Q3: 0.997,  Max: 1 | Total: 84573,  Min: 0,  Q1: 0.665,  Median: 0.971,  Q3: 0.997,  Max: 1 | Total: 579,  Min: 0,  Q1: 0.1665,  Median: 0.892,  Q3: 0.99,  Max: 1 |
| **A3** | Total: 20316,  Min: 0,  Q1: 0.736,  Median: 0.981,  Q3: 0.998,  Max: 1 | Total: 94182,  Min: 0,  Q1: 0.708,  Median: 0.979,  Q3: 0.998,  Max: 1 | Total: 778,  Min: 0,  Q1: 0.1385,  Median: 0.9075,  Q3: 0.993,  Max: 1 |

A summary of the evolutionary conservation of variants located at exonic splice junction regions is provided. Each cell contains the total count value along with the minimum (min), Q1, median (med), Q3, and maximum (max). Rows correspond to the six splice site positions of donors and acceptors, while the columns represent three different datasets: COSMIC, gnomAD >0 and <0.1%, and gnomAD ≥0.1%.
