# Supplementary material for: Synonymous and non-synonymous variants at splice junctions can disrupt splicing and are frequently linked to disease associated loss of function genes
Source: BMC Genomics. 2025 Dec 23;27:99. doi: 10.1186/s12864-025-12466-0 (PMC12838422; doi:10.1186/s12864-025-12466-0)
Supplement: Supplementary file 12 — Supplementary Material 12. Table S8 The AbSplice prediction score of all COSMIC and gnomAD-associated exonic variants in the splice junction [file 12864_2025_12466_MOESM12_ESM.docx]

**Table S8.** **The AbSplice prediction score of all COSMIC and gnomAD-associated exonic variants in the splice junction**

| **Site** | **All sites** | **COSMIC** | **gnomAD <0.1%** | **gnomAD >=0.1%** |
| --- | --- | --- | --- | --- |
| **d3** | Total: 215185,  Min: 0.01,  Q1: 0.019, Median: 0.0567, Q3: 0.141, Max: 0.479 | Total: 3934,  Min: 0.01,  Q1: 0.014, Median: 0.0259, Q3: 0.06,  Max: 0.417 | Total: 18014,  Min: 0.01,  Q1: 0.014, Median: 0.025, Q3: 0.056, Max: 0.416 | Total: 111,  Min: 0.01,  Q1: 0.012, Median: 0.02, Q3: 0.034, Max: 0.334 |
| **d2** | Total: 413618,  Min: 0.01,  Q1: 0.026, Median: 0.066, Q3: 0.174, Max: 0.479 | Total: 6827,  Min: 0.01,  Q1: 0.015, Median: 0.03, Q3: 0.076,  Max: 0.47 | Total: 34291,  Min: 0.01,  Q1: 0.016, Median: 0.031, Q3: 0.078, Max: 0.47 | Total: 212,  Min: 0.01,  Q1: 0.012, Median: 0.02, Q3: 0.045, Max: 0.369 |
| **d1** | Total: 1085809,  Min: 0.01,  Q1: 0.079, Median: 0.236, Q3: 0.302,  Max: 0.479 | Total: 25222,  Min: 0.01,  Q1: 0.058, Median: 0.177, Q3: 0.289,  Max: 0.479 | Total: 79670,  Min: 0.01,  Q1: 0.014, Median: 0.133, Q3: 00.27, Max: 0.479 | Total: 304,  Min: 0.01,  Q1: 0.032, Median: 0.06, Q3: 0.1495, Max: 0.368 |
| **a1** | Total: 310582,  Min: 0.01,  Q1: 0.017, Median: 0.041, Q3: 0.077, Max: 0.479 | Total: 8393,  Min: 0.01,  Q1: 0.014, Median: 0.025, Q3: 0.052,  Max: 0.416 | Total: 34583,  Min: 0.01,  Q1: 0.014, Median: 0.024, Q3: 0.05, Max: 0.479 | Total: 139,  Min: 0.01,  Q1: 0.012, Median: 0.017, Q3: 0.0345, Max: 0.262 |
| **a2** | Total: 77659,  Min: 0.01,  Q1: 0.013, Median: 0.024, Q3: 0.055, Max: 0.4695 | Total: 1724,  Min: 0.01,  Q1: 0.012, Median: 0.019, Q3: 0.04,  Max: 0.403 | Total: 7811,  Min: 0.01,  Q1: 0.012, Median: 0.019, Q3: 0.04, Max: 0.47 | Total: 60,  Min: 0.01,  Q1: 0.014, Median: 0.025, Q3: 0.0513, Max: 0.171 |
| **a3** | Total: 74810,  Min: 0.01,  Q1: 0.013, Median: 0.024, Q3: 0.047, Max: 0.41 | Total: 2074,  Min: 0.01,  Q1: 0.012, Median: 0.017, Q3: 0.032,  Max: 0.392 | Total: 10280,  Min: 0.01,  Q1: 0.013, Median: 0.02, Q3: 0.034, Max: 0.416 | Total: 81,  Min: 0.01,  Q1: 0.013, Median: 0.016, Q3: 0.0306, Max: 0.138 |

AbSplice scores are reported for six splice site types (d3, d2, d1, a1, a2, a3) across all variants, COSMIC, rare gnomAD (<0.1%), and common gnomAD (≥0.1%) datasets. Each entry includes total variant count, min, Q1, median, Q3, and max.
