# Supplementary material for: Synonymous and non-synonymous variants at splice junctions can disrupt splicing and are frequently linked to disease associated loss of function genes
Source: BMC Genomics. 2025 Dec 23;27:99. doi: 10.1186/s12864-025-12466-0 (PMC12838422; doi:10.1186/s12864-025-12466-0)
Supplement: Supplementary file 13 — Supplementary Material 13. Table S9 ClinVar assessment of exonic variants at the splice junction [file 12864_2025_12466_MOESM13_ESM.docx]

**Table S9.** **ClinVar assessment of exonic variants at the splice junction.**

**All**

| **Variant Impact** | **d3** | **d2** | **d1** | **a1** | **a2** | **a3** | **Total** |
| --- | --- | --- | --- | --- | --- | --- | --- |
| **Pathogenic** | 878 | 351 | 809 | 423 | 301 | 301 | **3063** |
| **Likely pathogenic** | 459 | 349 | 1018 | 511 | 340 | 221 | **2898** |
| **Benign** | 217 | 268 | 164 | 148 | 239 | 402 | **1438** |
| **Likely benign** | 1912 | 894 | 449 | 931 | 2485 | 4146 | **10817** |
| **Uncertain significance** | 6721 | 7719 | 6463 | 8483 | 5906 | 5709 | **41001** |
| **Others** | 474 | 724 | 884 | 595 | 502 | 558 | **3737** |
| **Total** | **10661** | **10305** | **9787** | **11091** | **9773** | **11337** | **62954** |

**COSMIC**

| **Variant Impact** | **d3** | **d2** | **d1** | **a1** | **a2** | **a3** | **Total** |
| --- | --- | --- | --- | --- | --- | --- | --- |
| **Pathogenic** | 22 | 20 | 109 | 30 | 26 | 25 | **232** |
| **Likely pathogenic** | 37 | 29 | 152 | 40 | 37 | 18 | **313** |
| **Benign** | 19 | 24 | 13 | 8 | 23 | 35 | **122** |
| **Likely benign** | 210 | 173 | 90 | 83 | 269 | 445 | **1270** |
| **Uncertain significance** | 849 | 1249 | 1020 | 734 | 692 | 711 | **5255** |
| **Others** | 99 | 189 | 200 | 68 | 86 | 109 | **751** |
| **Total** | **1236** | **1684** | **1584** | **963** | **1133** | **1343** | **7943** |

**gnomAD > 0 and <0.1%**

| **Variant Impact** | **d3** | **d2** | **d1** | **a1** | **a2** | **a3** | **Total** |
| --- | --- | --- | --- | --- | --- | --- | --- |
| **Pathogenic** | 44 | 55 | 245 | 64 | 35 | 50 | **493** |
| **Likely pathogenic** | 59 | 95 | 334 | 131 | 85 | 51 | **755** |
| **Benign** | 87 | 106 | 68 | 62 | 105 | 173 | **601** |
| **Likely benign** | 1278 | 679 | 320 | 691 | 1690 | 2845 | **7503** |
| **Uncertain significance** | 4625 | 5478 | 3865 | 5581 | 3951 | 3905 | **27405** |
| **Others** | 341 | 557 | 578 | 401 | 378 | 429 | **2684** |
| **Total** | **6434** | **6970** | **5410** | **6930** | **6244** | **7453** | **39441** |

**gnomAD >= 0.1%**

| **Variant Impact** | **d3** | **d2** | **d1** | **a1** | **a2** | **a3** | **Total** |
| --- | --- | --- | --- | --- | --- | --- | --- |
| **Pathogenic** | 1 | 2 | 0 | 0 | 0 | 0 | **3** |
| **Likely pathogenic** | 1 | 0 | 2 | 2 | 1 | 0 | **6** |
| **Benign** | 118 | 151 | 89 | 74 | 118 | 207 | **757** |
| **Likely benign** | 83 | 74 | 67 | 69 | 67 | 107 | **467** |
| **Uncertain significance** | 19 | 13 | 7 | 8 | 10 | 10 | **67** |
| **Others** | 20 | 28 | 22 | 29 | 27 | 24 | **150** |
| **Total** | **242** | **268** | **187** | **182** | **223** | **348** | **1450** |

The ClinVar evaluation of variants occurring at the last three nucleotide positions of acceptor and donor splice sites. Rows represent the ClinVar classification of variants into six main groups: pathogenic, likely pathogenic, benign, likely benign, uncertain significance, and others. Columns correspond to the six different splice site positions analyzed.
