# Supplementary material for: Synonymous and non-synonymous variants at splice junctions can disrupt splicing and are frequently linked to disease associated loss of function genes
Source: BMC Genomics. 2025 Dec 23;27:99. doi: 10.1186/s12864-025-12466-0 (PMC12838422; doi:10.1186/s12864-025-12466-0)
Supplement: Supplementary file 14 — Supplementary Material 14. Table S10 SpliceVarDB assessment of exonic variants at the splice junction [file 12864_2025_12466_MOESM14_ESM.docx]

**Table S10.** **SpliceVarDB assessment of exonic variants at the splice junction**

**All**

| **Site** | **Splice-altering** | **Normal** | **Low frequency** | **Conflicting** | **Total** |
| --- | --- | --- | --- | --- | --- |
| **d3** | 90 | 46 | 169 | 1 | **306** |
| **d2** | 172 | 60 | 202 | 1 | **435** |
| **d1** | 1045 | 53 | 471 | 1 | **1570** |
| **a1** | 155 | 77 | 230 | 4 | **466** |
| **a2** | 18 | 59 | 177 | 2 | **256** |
| **a3** | 12 | 79 | 192 | 0 | **283** |
| **Total** | **1492** | **374** | **1441** | **9** | **3316** |

**COSMIC**

| **Site** | **Splice-altering** | **Normal** | **Low frequency** | **Conflicting** | **Total** |
| --- | --- | --- | --- | --- | --- |
| **d3** | 66 | 6 | 54 | 0 | 126 |
| **d2** | 104 | 14 | 68 | 0 | 186 |
| **d1** | 684 | 5 | 265 | 0 | 954 |
| **a1** | 85 | 5 | 74 | 1 | 165 |
| **a2** | 8 | 9 | 29 | 0 | 46 |
| **a3** | 7 | 4 | 17 | 0 | 28 |
| **Total** | 954 | 43 | 507 | 1 | 1505 |

**gnomAD > 0 and < 0.1%**

| **Site** | **Splice-altering** | **Normal** | **Low frequency** | **Conflicting** | **Total** |
| --- | --- | --- | --- | --- | --- |
| **d3** | 38 | 37 | 106 | 1 | 182 |
| **d2** | 52 | 48 | 117 | 1 | 218 |
| **d1** | 306 | 46 | 180 | 0 | 532 |
| **a1** | 52 | 60 | 115 | 1 | 228 |
| **a2** | 14 | 52 | 103 | 0 | 169 |
| **a3** | 7 | 69 | 137 | 0 | 213 |
| **Total** | 469 | 312 | 758 | 3 | 1542 |

**gnomAD >= 0.1%**

| **Site** | **Splice-altering** | **Normal** | **Low frequency** | **Conflicting** | **Total** |
| --- | --- | --- | --- | --- | --- |
| **d3** | 0 | 1 | 5 | 0 | 6 |
| **d2** | 0 | 2 | 11 | 0 | 13 |
| **d1** | 3 | 2 | 4 | 0 | 9 |
| **a1** | 1 | 2 | 4 | 0 | 7 |
| **a2** | 0 | 0 | 4 | 0 | 4 |
| **a3** | 1 | 1 | 7 | 0 | 9 |
| **Total** | 5 | 8 | 35 | 0 | 48 |

The classification of variants from SpliceVarDB by their predicted impact on splicing (splice-altering, normal, low-frequency, conflicting) across six exonic splice site positions (d3, d2, d1, a1, a2, a3). For each position, counts are provided for all variants as well as those overlapping with the COSMIC database and gnomAD dataset stratified by population allele frequency (<0.1% and ≥0.1%).
