# Supplementary material for: Synonymous and non-synonymous variants at splice junctions can disrupt splicing and are frequently linked to disease associated loss of function genes
Source: BMC Genomics. 2025 Dec 23;27:99. doi: 10.1186/s12864-025-12466-0 (PMC12838422; doi:10.1186/s12864-025-12466-0)
Supplement: Supplementary file 15 — Supplementary Material 15. Table S11 Experimentally validated silent and missense variants affecting normal splicing [file 12864_2025_12466_MOESM15_ESM.pdf]

Table S11. Experimentally validated silent and missense variants affecting normal splicing

Gene	Chromosome	Position (hg38)	Reference allele	Alternate allele	Amino acid change	cDNA change	Splice site	Experiment RNAseq/ RT-PCR/ Mini gene assay/	Reference
ABCA3	16	2319581	C	T	p.K291K	c.G873A	d1	RT-PCR	[1]
ABCA4	1	94005441	C	T	p.K2049K	c.G6147A	d1	minigene	[2]
ABCA4	1	94023386	C	T	p.R1556K	c.G4667A	d1	minigene	[2]
ABCA4	1	94111579	C	T	p.C54Y	c.G161A	a1	minigene	[2]
ABCA4	1	94024955	T	C	p.S1545G	c.A4633G	d2	RT-PCR	[3]
ABCA4	1	94037350	C	T	p.G1203E	c.G3608A	a1	RT-PCR	[3]
ABCA4	1	94031778	C	G	p.Q1376H	c.G4128C	d1	RT-PCR	[3]
ABCC6	16	16154873	C	T	p.Q1347Q	c.G4041A	d1	RT-PCR	[4]
ABCC8	11	17453119	C	T	p.Q392Q	c.G1176A	d1	RT-PCR	[5]
ABCC8	11	17393697	C	T	p.A1537A	c.G4611A	d1	RT-PCR	[5]
AIMP1	4	106327564	G	A	p.V75M	c.G223A	d1	Minigene	[6]
ARID1B	6	157167185	G	T	p.A1009S	c.G3025T	d1	RNAseq	[7]
ARID2	12	45849775	A	T	p.A637A	c.A1911T	d2	RNAseq	[7]
ARPC1B	7	99391253	G	A	p.A261A	c.G783A	d1	cDNA sequencing	[8]
ASS1	9	130466726	T	G	p.V141G	c.T422G	a2	Minigene	[9]
ASXL1	20	32433917	G	A	p.R573R	c.G1719A	d1	RNAseq	[7]
ATM	11	108281168	G	A	p.K1192K	c.G3576A	d1	cDNA sequencing	[10]
ATM	11	108235834	G	C	p.E166Q	c.G496C	d1	RT-PCR	[11]
ATM	11	108281168	G	A	p.K1192K	c.G3576A	d1	RT-PCR	[12]
ATP6V0A4	7	138739540	C	T	p.P524P	c.G1572A	d1	minigene	[13]
ATP6V1B1	2	70959018	G	T	p.G123V	c.G368T	a1	minigene	[13]
ATP6V1B1	2	70959020	C	T	p.R124W	c.C370T	a3	minigene	[13]
ATP7B	13	51944109	C	T	p.E1081E	c.G3243A	d1	minigene	[14]
ATP7B	13	51946284	C	A	p.K942N	c.G2826T	d1	minigene	[14]
BAP1	3	52402604	T	A	p.E685V	c.A2054T	d3	RT-PCR	[15]
BBS1	11	66515586	G	A	p.R160Q	c.G479A	d1	RT-PCR	[16]
BCAP31	X	153723153	C	T	p.R31K	c.G92A	d1	RT-PCR	[17]

BLNK	10	96223826	C	T	p.E175E	c.G525A	d1	RT-PCR	[18]
BRAT1	7	2540979	C	T	p.T465T	c.G1395A	d1	RT-PCR	[19]
BRCA1	17	43047702	C	G	p.G1803A	c.G5408C	a2	mini-gene	[20]
BRCA1	17	43051063	C	T	p.D1778N	c.G5332A	d1	mini-gene	[20]
BRCA1	17	43051063	C	A	p.D1778Y	c.G5332T	d1	mini-gene	[20]
BRCA1	17	43067608	C	G	p.D1692H	c.G5074C	d1	mini-gene	[20]
BRCA1	17	43067608	C	T	p.D1692N	c.G5074A	d1	mini-gene	[20]
BRCA1	17	43067608	C	A	p.D1692Y	c.G5074T	d1	mini-gene	[20]
BRCA1	17	43067609	T	G	p.T1691T	c.A5073C	d2	RNAseq	[7]
BRCA1	17	43067694	A	T	p.M1663K	c.T4988A	a2	mini-gene	[20]
BRCA1	17	43067695	T	A	p.M1663L	c.A4987T	a1	mini-gene	[20]
BRCA1	17	43067610	G	C	p.T1691R	c.C5072G	d3	RT-PCR	[21]
BRCA1	17	43067610	G	T	p.T1691K	c.C5072A	d3	RT-PCR	[21]
BRCA1	17	43047703	C	A	p.G1803C	c.G5407T	a1	RT-PCR	[22]
BRCA1	17	43076488	C	T	p.R1495K	c.G4484A	d1	RT-PCR	[23]
BRCA1	17	43074331	C	T	p.E1559K	c.G4675A	d1	RT-PCR	[23]
BRCA2	13	32316527	G	A	p.D23N	c.G67A	d1	minigene	[24]
BRCA2	13	32326282	G	A	p.K172K	c.G516A	d1	minigene	[25]
BRCA2	13	32357929	G	C	p.R2602T	c.G7805C	d1	minigene	[26]
BRCA2	13	32363533	G	A	p.K2777K	c.G8331A	d1	minigene	[27]
BRCA2	13	32379913	G	A	p.P3039P	c.G9117A	d1	cDNA sequencing (cBROCA sequencing)	[28]
BRCA2	13	32329444	C	T	p.V211V	c.C633T	a2	RT-PCR	[21]
BRCA2	13	32329491	C	T	p.A227V	c.C680T	d2	RT-PCR	[29]
BRCA2	13	32346895	C	T	p.R2336C	c.C7006T	d2	RT-PCR	[21]
BRCA2	13	32379912	C	T	p.P3039L	c.C9116T	d2	RT-PCR	[21]
BRCA2	13	32344651	A	T	p.D2312V	c.A6935T	d3	RT-PCR	[21]
BRCA2	13	32356609	G	A	p.Q2539Q	c.G7617A	d1	RT-PCR	[21]
BRCA2	13	32325184	G	T	p.S142I	c.G425T	d1	RT-PCR	[30]
CATSPERG	19	38365061	G	A	p.V853M	c.G2557A	a1	MaPSY	[31]
CCDC129	7	31569809	G	C	p.R113T	c.G338C	d1	MaPSY	[31]
CDH23	10	71790413	G	A	p.G2017S	c.G6049A	d1	Minigene	[32]
CDKN2A	9	21974679	T	G	p.Q50P	c.A149C	d2	RT-PCR	[33]
CFTR	7	117531113	A	T	p.K163M	c.A488T	d2	Minigene	[34]

CFTR	7	117542016	G	T	p.D373Y	c.G1117T	a1	Minigene	[34]
CFTR	7	117542108	G	T	p.E403D	c.G1209T	d1	Minigene	[34]
CFTR	7	117611808	G	C	p.G1123R	c.G3367C	d1	Minigene	[34]
CFTR	7	117530899	G	A	p.E92K	c.G274A	a1	RT-PCR	[35]
CFTR	7	117606753	G	A	p.Q996Q	c.G2988A	d1	Minigene	[36]
CFTR	7	117642593	G	C	p.Q1291H	c.G3873C	d1	RT-PCR	[35]
CFTR	7	117627770	G	C	p.R1239S	c.G3717C	d1	RT-PCR	[35]
CFTR	7	117603782	G	C	p.G970R	c.G2908C	d1	RT-PCR	[35],[37], [38]
CFTR	7	117603782	G	A	p.G970S	c.G2908A	d1	RT-PCR	[35]
CHD7	8	60838076	G	T	p.V1452L	c.G4354T	a1	minigene	[39]
CLCN5	X	50070030	G	A	p.E35E	c.G105A	d1	Minigene	[40]
CLCNKB	1	16045686	G	A	p.A77T	c.G229A	d1	minigene	[41]
CLCNKB	1	16045686	G	C	p.A77P	c.G229C	d1	minigene	[41]
CLDN16	3	190408314	G	A	p.G198D	c.G593A	a1	Minigene	[42]
CLDN16	3	190408314	G	C	p.G198A	c.G593C	a1	Minigene	[42]
CLDN19	1	42738421	C	A	p.G130C	c.G388T	d1	Minigene	[43]
CNGB3	8	86739656	T	C	p.Q70Q	c.A210G	d2	Minigene	[44]
CNGB3	8	86668019	C	G	p.D215H	c.G643C	d1	Minigene	[44]
CNGB3	8	86647802	T	C	p.K330R	c.A989G	d2	Minigene	[44]
CNGB3	8	86644622	C	T	p.R352K	c.G1055A	d1	Minigene	[44]
CNGB3	8	86604093	C	G	p.S594T	c.G1781C	d1	Minigene	[44]
CNGB3	8	86578689	C	G	p.Q701H	c.G2103C	d1	Minigene	[44]
CNNM4	2	96797646	A	G	p.T560T	c.A1680G	d2	RNAseq	[7]
COCH	14	30879485	G	T	p.G211C	c.G631T	d1	Minigene	[45]
COL17A1	10	104060121	G	A	p.A380V	c.C1139T	d3	Minigene	[46]
COL17A1	10	104053920	C	T	p.G612R	c.G1834A	d1	Minigene	[46]
COL1A2	7	94408823	G	A	p.K264K	c.G792A	d1	minigene	[47]
COL1A2	7	94422957	G	A	p.G802S	c.G2404A	a1	minigene	[47]
COL27A1	9	114211026	G	A	p.P789P	c.G2367A	d1	RT-PCR	[48]
COL4A3	2	227253638	G	A	p.T255T	c.G765A	d1	Minigene, RT-PCR	[49, 50]
COL4A4	2	227103144	C	T	p.K290K	c.G870A	d1	mRNA Analysis, minigene	[51]
COL4A4	2	227108581	C	T	p.P245P	c.G735A	d1	mRNA Analysis, minigene	[51]

COL4A5	X	108586747	G	A	p.G389R	c.G1165A	d1	Minigene	[52]
COL4A5	X	108591644	G	A	p.G475S	c.G1423A	d1	Minigene	[52]
COL4A5	X	108598870	G	T	p.G650C	c.G1948T	d1	Minigene	[52]
COL4A5	X	108598870	G	A	p.G650S	c.G1948A	d1	Minigene	[52]
COL4A5	X	108601988	A	G	p.K715K	c.A2145G	d2	Minigene	[53]
COL4A5	X	108603061	G	T	p.K748N	c.G2244T	d1	Minigene	[52]
COL4A5	X	108606891	A	T	p.K798N	c.A2394T	d2	Minigene	[53]
COL4A5	X	108606891	A	G	p.K798K	c.A2394G	d2	Minigene	[53]
COL4A5	X	108615024	G	A	p.G837S	c.G2509A	d1	Minigene	[52]
COL4A5	X	108620426	G	C	p.G893R	c.G2677C	d1	Minigene	[52]
COL4A5	X	108620426	G	A	p.G893S	c.G2677A	d1	Minigene	[52]
COL4A5	X	108621892	G	C	p.G923R	c.G2767C	d1	Minigene	[52]
COL4A5	X	108655457	G	A	p.G1125R	c.G3373A	d1	Minigene	[52]
COL4A5	X	108668504	G	A	p.G1264R	c.G3790A	d1	Minigene	[52]
COL4A5	X	108677633	G	C	p.Q1308H	c.G3924C	d1	Minigene	[52]
COL4A5	X	108680751	G	A	p.G1333S	c.G3997A	d1	Minigene	[52]
COL4A5	X	108680956	G	C	p.G1357R	c.G4069C	d1	Minigene	[52]
COL4A5	X	108680956	G	A	p.G1357S	c.G4069A	d1	Minigene	[52]
COL4A5	X	108692925	G	A	p.R1563Q	c.G4688A	d1	Minigene	[52]
COL4A5	X	108695437	C	T	p.F1658F	c.C4974T	d3	Minigene	[53]
COL4A5	X	108695438	A	G	p.S1659G	c.A4975G	d2	Minigene	[53]
COL4A5	X	108695439	G	A	p.S1659N	c.G4976A	d1	Minigene	[52]
COL4A5	X	108621803	G	C	p.G893A	c.G2678C	a1	Minigene	[54]
COL4A5	X	108624236	G	A	p.G973D	c.G2918A	a1	Minigene	[54]
COL5A2	2	189062865	C	T	p.P659P	c.G1977A	d1	RT-PCR	[55]
CRB1	1	197434706	G	A	p.C948Y	c.G2843A	a1	cDNA sequencing	[56]
CYP27B1	12	57765211	C	T	p.G197D	c.G590A	a1	minigene	[57]
CYP27B1	12	57765211	C	T	p.G197D	c.G590A	a1	RT-PCR	[58]
DES	2	219420346	G	C	p.E245D	c.G735C	d1	RT-PCR	[59]
DNMT3A	2	25243899	T	A	p.T645T	c.A1935T	d2	RNAseq	[7]
DSPP	4	87612107	T	C	p.V18V	c.T54C	a3	Minigene	[60]
DSPP	4	87612106	T	A	p.V18D	c.T53A	a2	Minigene	[60]
DSPP	4	87610957	C	A	p.P17T	c.C49A	d3	Minigene	[60]
DSPP	4	87612106	T	G	p.V18G	c.T53G	a2	Minigene	[60]
DSPP	4	87612106	T	C	p.V18A	c.T53C	a2	Minigene	[60]

DSPP	4	87612107	T	A	p.V18V	c.T54A	a3	Minigene	[60]
DSPP	4	87610957	C	T	p.P17S	c.C49T	d3	Minigene	[60]
DYSF	2	71513242	G	A	p.G155R	c.G463A	a3	minigene	[61]
EFTUD2	17	44853390	C	T	p.A823T	c.G2467A	a1	minigene	[62]
EFTUD2	17	44859905	C	G	p.K620N	c.G1860C	d1	minigene	[62]
EFTUD2	17	44859905	C	A	p.K620N	c.G1860T	d1	minigene	[62]
EFTUD2	17	44867807	C	G	p.Q383H	c.G1149C	d1	minigene	[62]
EFTUD2	17	44879556	C	A	p.G234G	c.G702T	d1	cDNA sequencing	[63]
EP300	22	41147946	G	A	p.P747P	c.G2241A	d1	RNAseq	[7]
EPG5	18	45954395	T	C	p.Q336R	c.A1007G	d2	RT-PCR	[64]
EYA4	6	133481599	G	T	p.E369D	c.G1107T	d1	Minigene	[65]
EZH2	7	148826471	T	C	p. H297R	c.A890G	d3	minigene	[66]
EZH2	7	148826631	A	C	p.Y244D	c.T730G	a2	minigene	[66]
F5	1	169523904	C	T	p.G1930D	c.G5789A	a1	Minigene	[67]
F8	X	154987305	C	T	p.G201E	c.G602A	a1	Minigene	[68]
F8	X	154987238	T	A	p.E223D	c.A669T	d2	Minigene	[68]
F8	X	154956957	C	T	p.Q584Q	c.G1752A	d1	Minigene	[69]
F8	X	154987237	C	T	p.G224R	c.G670A	d1	Minigene	[69]
F8	X	154987237	C	A	p.G224W	c.G670T	d1	Minigene	[68]
F8	X	154987238	T	C	p.E223E	c.A669G	d2	Minigene	[68]
F8	X	154969331	C	T	p.D337N	c.G1009A	d1	Minigene	[69]
F8	X	154899866	C	T	p.K2091K	c.G6273A	d1	Minigene	[70]
F9	X	139530851	A	G	p.T29T	c.A87G	d2	RT-PCR	[71]
FAH	15	80173143	A	G	p.Q279R	c.A836G	d2	Minigene	[72]
FANCL	2	58160108	C	T	p.K364K	c.G1092A	d1	RT-PCR	[73]
FBN1	15	48445504	T	C	p.D1930G	c.A5789G	a1	RT-PCR	[22]
FBN1	15	48481656	T	C	p.T1321T	c.A3963G	d2	RT-PCR	[22]
FBN1	15	48513549	C	T	p.D530N	c.G1588A	d1	RT-PCR	[22]
FBN1	15	48485374	C	T	p.D1238N	c.G3712A	d1	RT-PCR	[22]
FECH	18	57571392	C	G	p.A155P	c.G463C	d1	cDNA sequencing	[74]
FLAD1	1	154990526	C	T	p.L518L	c.C1552T	d3	RNAseq	[7]
FMR1	X	147932595	G	A	p.E267E	c.G801A	d1	RT-PCR	[75]
GAA	17	80105132	G	T	p.T182T	c.G546T	d1	RT-PCR	[76]
GALT	9	34648762	G	A	p.E230K	c.G688A	a1	RT-PCR	[77]
GCK	7	44146463	C	T	p.S340N	c.G1019A	d1	minigene	[78]

GCK	7	44146463	C	G	p.S340T	c.G1019C	d1	minigene	[78]
GCK	7	44149760	C	T	p.G227S	c.G679A	d1	minigene	[78]
GCK	7	44149760	C	G	p.G227R	c.G679C	d1	minigene	[78]
GCK	7	44149969	C	A	p.G193G	c.G579T	d1	minigene	[78]
GCK	7	44150064	C	T	p.G162S	c.G484A	a1	minigene	[78]
GCK	7	44153301	C	T	p.E70K	c.G208A	d1	minigene	[78]
GCK	7	44153301	C	G	p.E70Q	c.G208C	d1	minigene	[78]
GCK	7	44152424	T	G	p.E69D	c.A207C	a2	Minigene	[79]
GMPR	6	16274496	G	C	p.G183R	c.G547C	d1	RT-PCR	[80]
GSAP	7	77377287	T	G	p.K227T	c.A680C	d2	MaPSY	[31]
HFM1	1	91276984	C	T	p.C1157Y	c.G3470A	d3	Minigene	[81]
HNF1A	12	120997665	G	A	p.A501T	c.G1501A	d1	minigene	[78]
HNF1A	12	120999389	G	A	p.Q541Q	c.G1623A	d1	minigene	[78]
HNF4A	20	44413800	G	A	p.Q142Q	c.G426A	d1	minigene	[78]
HNF4A	20	44424254	G	C	p.G355R	c.G1063C	d1	minigene	[78]
HSD17B3	9	96240907	C	T	p.V225M	c.G673A	a1	RT-PCR	[82]
IDUA	4	1004111	A	T	p.P609P	c.A1827T	d2	MaPSY	[31]
IFT74	9	26978263	G	A	p.G86S	c.G256A	d1	RT-PCR	[83]
IFT74	9	26978263	G	A	p.G86S	c.G256A	d1	RT-PCR	[84]
IL2RG	X	71108599	C	T	p.R285Q	c.G868A	d1	RT-PCR	[85]
IRF2	4	184418533	T	C	p.K121K	c.A363G	d2	RNAseq	[7]
JAG1	20	10647960	C	G	p.V574L	c.G1720C	d1	cDNA sequencing	[86]
JAK3	19	17835924	C	A	p.L638L	c.G1914T	d1	minigene	[87]
KIAA0586	14	58458545	G	A	p.Q605Q	c.G1815A	d1	RT-PCR	[88]
KIF11	10	92649986	G	A	p.P974P	c.G2922A	d1	minigene	[89]
KMT2C	7	152207301	T	C	p.P1280P	c.A3840G	d2	RNAseq	[7]
LAMB3	1	209633070	C	T	p.E210K	c.G628A	d1	RT-PCR	[90]
LCA5	6	79492551	C	T	p.A319T	c.G955A	d1	cDNA sequence	[91]
LMNA	1	156135312	G	C	p.Q312H	c.G936C	d1	minigene	[92]
LMNA	1	156135312	G	C	p.Q312H	c.G936C	d1	Minigene	[93]
LPIN2	18	2929065	C	T	p.R517H	c. G1550A	d1	RT-PCR	[94]
LRP5	11	68403483	G	T	p.V529L	c.G1585T	a1	Minigene	[21]
LRRK1	15	100973967	G	A	p.K87K	c.G261A	d1	RT-PCR	[95]
MEIOB	16	1839255	C	T	p.T406T	c.G1218A	d1	Minigene	[96]
MFSD8	4	127921524	C	T	p.Q450Q	c.G1350A	d1	RNAseq	[7]

MLH1	3	37042331	G	A	p.S577S	c.G1731A	d1	RT-PCR	[97, 98]
MLH1	3	37049017	G	A	p.Q701Q	c.G2103A	d1	minigene	[99]
MLH1	3	37042331	G	A	p.S577S	c.G1731A	d1	RT-PCR	[100]
MYH9	22	36295505	C	G	p.R1162T	c.G3485C	d1	RT-PCR	[101]
MYO7A	11	77181589	G	A	p.E968E	c.G2904A	d1	minigene	[102]
MYSM1	1	58689038	C	T	p.L133L	c.G399A	d1	RT-PCR	[103]
NCF2	1	183567204	C	G	p.Q285H	c.G855C	d1	RT-PCR	[104]
NCKAP1L	12	54532250	G	C	p.K954N	c.G2862C	d1	MaPSY	[31]
NDUFS2	1	161212480	G	A	p.K372K	c.G1116A	d1	RNAseq	[7]
NF1	17	31258501	A	G	p.K1423R	c.A4268G	d2	cDNA sequencing	[105]
NF1	17	31327839	G	A	p.R1849Q	c.G5546A	d1	cDNA sequencing	[106]
NIPBL	5	37020877	G	A	p.Q1776Q	c.G5328A	d1	RT-PCR	[107]
NPC1	18	23539813	C	T	p.N931N	c.C2793T	d3	Minigene	[108]
NR5A1	9	124503079	C	A	p.A82S	c.G244T	d1	Minigene	[109]
OTC	X	38381429	G	A	p.R129H	c.G386A	d1	Minigene	[110]
P2RX1	17	3898009	C	T	p.A378A	c.G1134A	d1	MaPSY	[31]
PALB2	16	23624093	A	G	p.V917A	c.T2750C	a2	Minigene	[111]
PALB2	16	23607866	G	A	p.G1116G	c.C3348T	d3	Minigene	[111]
PALB2	16	23624009	C	G	p.R945T	c.G2834C	d1	Minigene	[111]
PALB2	16	23629641	T	G	p.Q838P	c.A2513C	d2	Minigene	[111]
PALB2	16	23641111	T	C	p.K16R	c.A47G	d2	Minigene	[111]
PALB2	16	23641110	C	T	p.K16K	c.G48A	d1	Minigene	[111]
PATL2	15	44669563	C	A	p.D293Y	c.G877T	a1	RT-PCR	[112]
PAX6	11	31802705	T	G	p.Q47P	c.A140C	d2	Minigene	[113]
PAX6	11	31802704	C	T	p.Q47Q	c.G141A	d1	Minigene	[113]
PAX6	11	31794631	T	C	p.K227K	c.A681G	d2	Minigene	[113]
PAX6	11	31794630	C	T	p.E228K	c.G682A	d1	Minigene	[113]
PAX6	11	31794033	T	C	p.Q255R	c.A764G	d2	Minigene	[113]
PAX6	11	31794032	C	A	p.Q255H	c.G765T	d1	Minigene	[113]
PAX6	11	31794032	C	G	p.Q255H	c.G765C	d1	Minigene	[113]
PAX6	11	31790710	C	T	p.G395R	c.G1183A	d1	Minigene	[113]
PAX6	11	31802705	T	C	p.Q47R	c.A140G	d2	Minigene	[114]
PGK1	X	78125051	G	A	p.G372S	c.G1114A	d1	RT-PCR	[115]
PHEX	X	22212958	G	C	p.R567P	c.G1700C	d1	minigene	[116]
PHF21A	11	45948886	C	T	p.G429S	c.G1285A	d1	Minigene	[117]

PKD1	16	2092954	C	T	p.R3719Q	c.G11156A	d1	Minigene	[118]
PKD2	4	88052158	G	A	p.K572K	c.G1716A	d1	RT-PCR	[119]
PLG	6	160737006	A	C	p.R601R	c.A1801C	d2	RNAseq	[7]
PNKP	19	49867054	C	G	p.V51L	c.G151C	d1	RT-PCR	[120]
POLR3A	10	77984205	C	T	p.E1112E	c.G3336A	d1	RT-PCR	[121]
POMT2	14	77279823	C	G	p.G631R	c.G1891C	d1	Minigene	[122]
POT1	7	124825252	C	T	p.D598N	c.G1792A	d1	RT-PCR	[123]
POU1F1	3	87273347	C	T	p.G98S	c.G292A	d1	Minigene	[124]
POU1F1	3	87273347	C	G	p.G98R	c.G292C	d1	Minigene	[124]
POU1F1	3	87273347	C	A	p.G98C	c.G292T	d1	Minigene	[124]
POU1F1	3	87273348	T	G	p.A97A	c.A291C	d2	Minigene	[124]
POU1F1	3	87273348	T	C	p.A97A	c.A291G	d2	Minigene	[124]
POU1F1	3	87273348	T	A	p.A97A	c.A291T	d2	Minigene	[124]
POU1F1	3	87273349	G	A	p.A97V	c.C290T	d3	Minigene	[124]
POU1F1	3	87273495	G	C	p.V48V	c.C144G	a2	Minigene	[124]
POU1F1	3	87273496	A	T	p.V48D	c.T143A	a1	Minigene	[124]
POU1F1	3	87273496	A	G	p.V48A	c.T143C	a1	Minigene	[124]
POU1F1	3	87273496	A	C	p.V48G	c.T143G	a1	Minigene	[124]
PPOX	1	161169660	G	T	p.V270L	c.G808T	a1	RT-PCR	[125]
PPP6C	9	125171085	C	T	p.Q57Q	c.G171A	d1	RNAseq	[7]
PRKDC	8	47778459	C	T	p.Q3951Q	c.G11853A	d1	RNAseq	[7]
PRKDC	8	47828168	C	T	p.Q2859Q	c.G8577A	d1	RNAseq	[7]
PTEN	10	87961118	G	A	p.K342K	c.G1026A	d1	RNAseq	[7]
RAD50	5	132604046	G	A	p.V842I	c.G2524A	d1	RT-PCR	[126]
RAD51B	14	68411527	G	A	p.Q319Q	c.G957A	d1	RNAseq	[7]
RAD51D	17	35119532	C	T	p.V28M	c.G82A	d1	RT-PCR	[127]
RGS7	1	240801455	C	T	p.V471V	c.G1413A	d1	MaPSY	[31]
RHD	1	25301521	C	T	p.G212G	c.C636T	a2	Minigene	[128]
RHD	1	25306729	T	C	p.I358T	c.T1073C	d1	Minigene	[128]
RHD	1	25306597	G	T	p.G314V	c.G941T	a2	Minigene	[128]
RHD	1	25284574	T	C	p.V50V	c.T150C	a2	Minigene	[128]
RHD	1	25303458	C	T	p.P313L	c.C938T	d2	Minigene	[128]
RHD	1	25290790	A	G	p.N162S	c.A485G	d2	Minigene	[128]
RHD	1	25301685	A	T	p.K267M	c.A800T	d2	Minigene	[128]

RHD	1	25321889	G	A	p.G385D	c.G1154A	a1	Minigene	[129]
RHD	1	25321889	G	C	p.G385A	c.G1154C	a1	Minigene	[129]
RHD	1	25321889	G	T	p.G385V	c.G1154T	a1	Minigene	[129]
RPE65	1	68431282	C	A	p.R446S	c.G1338T	d1	Minigene	[130]
RPGR	X	38323399	C	T	p.G52R	c.G154A	d1	RT-PCR	[131]
SBF2	11	10029765	C	T	p.Q171Q	c.G513A	d1	RNAseq	[7]
SCN1A	2	165994146	C	T	p.G1618S	c.G4852A	d1	midigene	[132]
SCN1A	2	165994414	G	C	p.N1528K	c.C4584G	a3	Minigene	[124]
SCN1A	2	165998038	C	T	p.K1492K	c.G4476A	d1	Minigene	[124]
SCN1A	2	165999776	C	A	p.A1429S	c.G4285T	a1	Minigene	[124]
SCN1A	2	166041231	C	T	p.L805L	c.G2415A	d1	Minigene	[124]
SCN1A	2	166045043	C	A	p.Q554H	c.G1662T	d1	Minigene	[124]
SCN1A	2	166045043	C	T	p.Q554Q	c.G1662A	d1	Minigene	[124]
SCN1A	2	166045044	T	C	p.Q554R	c.A1661G	d2	Minigene	[124]
SGCA	17	50167487	G	A	p.A53T	c.G157A	d1	RT-PCR	[133]
SLC12A3	16	56870096	G	T	p.G201V	c.G602T	a1	minigene	[134]
SLC12A3	16	56870096	G	A	p.G201D	c.G602A	a1	minigene	[134]
SLC12A3	16	56880253	G	A	p.A523T	c.G1567A	d1	minigene	[134]
SLC12A3	16	56885364	G	A	p.R642H	c.G1925A	d1	minigene	[134]
SLC12A3	16	56893054	G	C	p.G850R	c.G2548C	d1	minigene	[134]
SLC12A3	16	56894531	G	C	p.G850A	c.G2549C	d1	minigene	[134]
SLC40A1	2	189563584	C	T	p.G468S	c.G1402A	a3	minigene	[135]
SLC40A1	2	189572846	G	A	p.L129L	c.C387T	d1	Minigene	[21]
SLC5A2	16	31485730	C	T	p.A102V	c.C305T	a2	Minigene	[136]
SLC5A2	16	31488038	G	C	p.V296L	c.G886C	a1	Minigene	[136]
SLC5A2	16	31488490	G	A	p.G377S	c.G1129A	d1	Minigene	[136]
SMN1	5	70076521	G	C	p.G279R	c.G835C	a1	Minigene	[137]
SPINK1	5	147829599	C	T	p.E29E	c.G87A	d1	FLGSA	[138]
SPINK1	5	147829599	C	G	p.E29D	c.G87C	d1	FLGSA	[138]
SPINK1	5	147829599	C	A	p.E29D	c.G87T	d1	FLGSA	[138]
SPINK1	5	147829600	T	C	p.E29G	c.A86G	d2	FLGSA	[138]
SPINK1	5	147829600	T	A	p.E29V	c.A86T	d2	FLGSA	[138]
SPINK1	5	147829630	C	G	p.G19A	c.G56C	a1	FLGSA	[138]
SPINK1	5	147829630	C	A	p.G19V	c.G56T	a1	FLGSA	[138]
SPINK1	5	147831523	C	T	p.G19S	c.G55A	d1	FLGSA	[138]

SPINK1	5	147831523	C	G	p.G19R	c.G55C	d1	FLGSA	[138]
SPINK1	5	147831523	C	A	p.G19C	c.G55T	d1	FLGSA	[138]
SYNGAP1	6	33432252	G	A	p.S129S	c.G387A	d1	MaPSY	[31]
TCF4	18	55261466	G	A	p.S330S	c.G990A	d1	MaPSY	[31]
TCIRG1	11	68043497	G	A	p.T210T	c.G630A	d1	Minigene	[139]
TMPRSS15	21	18326432	C	T	p.E641K	c.G1921A	d1	Minigene	[140]
TNRC6B	22	40273600	G	A	p.K1047K	c.G3141A	d1	Minigene	[141]
TNRC6C	17	78071165	G	A	p.P953P	c.G2859A	d1	MaPSY	[31]
TP53	17	7673535	C	T	p.Q331Q	c.G993A	d1	minigene	[142, 143]
TP53	17	7674181	C	G	p.S261T	c.G782C	d1	RNAseq	[7]
TP53	17	7674859	C	T	p.E224E	c.G672A	d1	minigene	[142, 144] [145]
TP53	17	7674859	C	A	p.E224D	c.G672T	d1	RNAseq	[7, 145]
TP53	17	7674970	A	T	p.G187G	c.T561A	a2	minigene	[142]
TP53	17	7674970	A	G	p.G187G	c.T561C	a2	minigene	[142]
TP53	17	7674970	A	C	p.G187G	c.T561G	a2	minigene	[142, 144]
TP53	17	7675053	C	G	p.G187R	c.G559C	d1	RNAseq	[7]
TP53	17	7675994	C	T	p.T125T	c.G375A	d1	minigene	[143, 146]
TP53	17	7675994	C	G	p.T125T	c.G375C	d1	minigene	[144, 146]
TP53	17	7675994	C	A	p.T125T	c.G375T	d1	minigene	[142, 144, 146]
TP53	17	7675995	G	A	p.T125M	c.C374T	d2	Minigene	[146]
TP53	17	7675995	G	C	p.T125R	c.C374G	d2	Minigene	[146]
TP53	17	7675996	T	C	p.T125A	c.A373G	d3	Minigene	[146]
TP53	17	7675996	T	G	p.T125P	c.A373C	d3	Minigene	[146, 147]
TREM2	6	41161263	C	T	p.D131N	c.G391A	d1	Minigene	[148]
TRMU	22	46355587	A	G	p.L339L	c.A1017G	d2	RNAseq	[7]
TTC8	14	88872452	G	C	p.Q449H	c.G1347C	d1	cDNA sequencing	[149]

U2AF2	19	55662618	G	T	p.E201D	c.G603T	d1	RT-PCR	[150]
UPF3B	X	119841735	C	A	p.Q208H	c.G624T	d1	RT-PCR	[151]
VWF	12	5976111	C	T	p.S2479S	c.G7437A	d1	cDNA sequencing	[152]
VWF	12	6072331	C	T	p.C370Y	c.G1109A	d1	cDNA sequencing	[152]
ZFN780B	19	40047375	C	T	p.D78N	c.G232A	d1	MaPSY	[31]

List of references

1. Zhang W, Liu Z, Lin Y, Wang R, Xu J, He Y, Zhang F, Wu L, Chen D: **A novel synonymous ABCA3 variant identified in a Chinese family with lethal neonatal respiratory failure.** *BMC Med Genomics* 2021, **14:**256.
2. Fadaie Z, Khan M, Del Pozo-Valero M, Cornelis SS, Ayuso C, Cremers FPM, Roosing S, The Abca Study G: **Identification of splice defects due to noncanonical splice site or deep-intronic variants in ABCA4.** *Hum Mutat* 2019, **40:**2365-2376.
3. Khan M, Cornelis SS, Khan MI, Elmelik D, Manders E, Bakker S, Derks R, Neveling K, van de Vorst M, Gilissen C, et al: **Cost-effective molecular inversion probe-based ABCA4 sequencing reveals deep-intronic variants in Stargardt disease.** *Hum Mutat* 2019, **40:**1749-1759.
4. Contro G, Tallerico R, Dattilo V, Fabiani F, Enzo MV, Hladnik U, Dastoli S, Nistico SP, Colao E, Perrotti N, Iuliano R: **A novel ABCC6 variant causative of pseudoxanthoma elasticum.** *Hum Genome Var* 2019, **6:**30.
5. Saint-Martin C, Cauchois-Le Miere M, Rex E, Soukarieh O, Arnoux JB, Buratti J, Bouvet D, Frebourg T, Gaildrat P, Shyng SL, et al: **Functional characterization of ABCC8 variants of unknown significance based on bioinformatics predictions, splicing assays, and protein analyses: Benefits for the accurate diagnosis of congenital hyperinsulinism.** *Hum Mutat* 2021, **42:**408-420.
6. Morais S, Leal Loureiro J, Brandao E, Sequeiros J, Stevanin G, Santos M: **Hereditary Spastic Paraplegia Linked to Abnormal Splicing From an AIMP1 Missense Variant.** *Clin Genet* 2025, **107:**668-672.
7. Palmisano A, Vural S, Zhao Y, Sonkin D: **MutSpliceDB: A database of splice sites variants with RNA-seq based evidence on effects on splicing.** *Hum Mutat* 2021, **42:**342-345.
8. Papadatou I, Marinakis N, Botsa E, Tzanoudaki M, Kanariou M, Orfanou I, Kanaka-Gantenbein C, Traeger-Synodinos J, Spoulou V: **Case Report: A Novel Synonymous ARPC1B Gene Mutation Causes a Syndrome of Combined Immunodeficiency, Asthma, and Allergy With Significant Intrafamilial Clinical Heterogeneity.** *Front Immunol* 2021, **12:**634313.
9. Kimani JK, Wei T, Chol K, Li Y, Yu P, Ye S, Huang X, Qi M: **Functional analysis of novel splicing and missense mutations identified in the ASS1 gene in classical citrullinemia patients.** *Clin Chim Acta* 2015, **438:**323-329.

10. Teraoka SN, Telatar M, Becker-Catania S, Liang T, Onengut S, Tolun A, Chessa L, Sanal O, Bernatowska E, Gatti RA, Concannon P: **Splicing defects in the ataxia-telangiectasia gene, ATM: underlying mutations and consequences.** *Am J Hum Genet* 1999, **64:**1617-1631.
11. Shimazaki H, Kobayashi J, Sugaya R, Nakano I, Fujimoto S: **Late-onset autosomal recessive cerebellar ataxia and neuropathy with a novel splicing mutation in the ATM gene.** *J Integr Neurosci* 2020, **19:**125-129.
12. Asadollahi R, Britschgi C, Joset P, Oneda B, Schindler D, Meier UR, Rauch A: **Severe reaction to radiotherapy provoked by hypomorphic germline mutations in ATM (ataxia-telangiectasia mutated gene).** *Mol Genet Genomic Med* 2020, **8:**e1409.
13. Zhang R, Chen Z, Song Q, Wang S, Liu Z, Zhao X, Shi X, Guo W, Lang Y, Bottillo I, Shao L: **Identification of seven exonic variants in the SLC4A1, ATP6V1B1, and ATP6V0A4 genes that alter RNA splicing by minigene assay.** *Hum Mutat* 2021, **42:**1153-1164.
14. Zhou X, Zhou W, Wang C, Wang L, Jin Y, Jia Z, Liu Z, Zheng B: **A Comprehensive Analysis and Splicing Characterization of Naturally Occurring Synonymous Variants in the ATP7B Gene.** *Front Genet* 2020, **11:**592611.
15. Morrison A, Chekaluk Y, Bacares R, Ladanyi M, Zhang L: **BAP1 missense mutation c.2054 A>T (p.E685V) completely disrupts normal splicing through creation of a novel 5' splice site in a human mesothelioma cell line.** *PLoS One* 2015, **10:**e0119224.
16. Breuel S, Vorm M, Brauer AU, Owczarek-Lipska M, Neidhardt J: **Combining Engineered U1 snRNA and Antisense Oligonucleotides to Improve the Treatment of a BBS1 Splice Site Mutation.** *Mol Ther Nucleic Acids* 2019, **18:**123-130.
17. Kao HJ, Chiang HL, Chen HH, Fan PC, Tu YF, Chou YY, Hwu WL, Lin CL, Kwok PY, Lee NC: **De novo mutation and skewed X-inactivation in girl with BCAP31-related syndrome.** *Hum Mutat* 2020, **41:**1775-1782.
18. Li N, Wu J, Wu Y, Xu Y, Yao R, Li G, Zhang J, Zhou Y, Yin L, Yin Y, et al: **Further delineation of primary B cell immunodeficiency caused by novel variants of the BLNK gene in two Chinese patients.** *Clin Immunol* 2020, **214:**108387.
19. Nuovo S, Baglioni V, De Mori R, Tardivo S, Caputi C, Ginevrino M, Micalizzi A, Masuelli L, Federici G, Casella A, et al: **Clinical variability at the mild end of BRAT1-related spectrum: Evidence from two families with genotype-phenotype discordance.** *Hum Mutat* 2022, **43:**67-73.
20. Ahlborn LB, Dandanell M, Steffensen AY, Jonson L, Nielsen FC, Hansen TV: **Splicing analysis of 14 BRCA1 missense variants classifies nine variants as pathogenic.** *Breast Cancer Res Treat* 2015, **150:**289-298.
21. Leman R, Gaildrat P, Le Gac G, Ka C, Fichou Y, Audrezet MP, Caux-Moncoutier V, Caputo SM, Boutry-Kryza N, Leone M, et al: **Novel diagnostic tool for prediction of variant spliceogenicity derived from a set of 395 combined in silico/in vitro studies: an international collaborative effort.** *Nucleic Acids Res* 2018, **46:**7913-7923.
22. Wai HA, Lord J, Lyon M, Gunning A, Kelly H, Cibin P, Seaby EG, Spiers-Fitzgerald K, Lye J, Ellard S, et al: **Correction: Blood RNA analysis can increase clinical diagnostic rate and resolve variants of uncertain significance.** *Genet Med* 2020, **22:**1129.

23. Wangensteen T, Felde CN, Ahmed D, Maehle L, Ariansen SL: **Diagnostic mRNA splicing assay for variants in BRCA1 and BRCA2 identified two novel pathogenic splicing aberrations.** *Hered Cancer Clin Pract* 2019, **17:**14.
24. Fraile-Bethencourt E, Valenzuela-Palomo A, Diez-Gomez B, Goina E, Acedo A, Buratti E, Velasco EA: **Mis-splicing in breast cancer: identification of pathogenic BRCA2 variants by systematic minigene assays.** *J Pathol* 2019, **248:**409-420.
25. Hansen TV, Steffensen AY, Jonson L, Andersen MK, Ejlertsen B, Nielsen FC: **The silent mutation nucleotide 744 G --> A, Lys172Lys, in exon 6 of BRCA2 results in exon skipping.** *Breast Cancer Res Treat* 2010, **119:**547-550.
26. Bonnet C, Krieger S, Vezain M, Rousselin A, Tournier I, Martins A, Berthet P, Chevrier A, Dugast C, Layet V, et al: **Screening BRCA1 and BRCA2 unclassified variants for splicing mutations using reverse transcription PCR on patient RNA and an ex vivo assay based on a splicing reporter minigene.** *Journal of Medical Genetics* 2008, **45:**438-446.
27. Sanz DJ, Acedo A, Infante M, Duran M, Perez-Cabornero L, Esteban-Cardenosa E, Lastra E, Pagani F, Miner C, Velasco EA: **A high proportion of DNA variants of BRCA1 and BRCA2 is associated with aberrant splicing in breast/ovarian cancer patients.** *Clin Cancer Res* 2010, **16:**1957-1967.
28. Peelen T, van Vliet M, Bosch A, Bignell G, Vasen HF, Klijn JG, Meijers-Heijboer H, Stratton M, van Ommen GJ, Cornelisse CJ, Devilee P: **Screening for BRCA2 mutations in 81 Dutch breast-ovarian cancer families.** *Br J Cancer* 2000, **82:**151-156.
29. de Garibay GR, Acedo A, Garcia-Casado Z, Gutierrez-Enriquez S, Tosar A, Romero A, Garre P, Llort G, Thomassen M, Diez O, et al: **Capillary electrophoresis analysis of conventional splicing assays: IARC analytical and clinical classification of 31 BRCA2 genetic variants.** *Hum Mutat* 2014, **35:**53-57.
30. Nix P, Mundt E, Coffee B, Goossen E, Warf BM, Brown K, Bowles K, Roa B: **Interpretation of BRCA2 Splicing Variants: A Case Series of Challenging Variant Interpretations and the Importance of Functional RNA Analysis.** *Fam Cancer* 2022, **21:**7-19.
31. Rhine CL, Neil C, Wang J, Maguire S, Buerer L, Salomon M, Meremikwu IC, Kim J, Strande NT, Fairbrother WG: **Massively parallel reporter assays discover de novo exonic splicing mutants in paralogs of Autism genes.** *PLoS Genet* 2022, **18:**e1009884.
32. Chen B, Zhang S, Tian YA, Liu HF, Liu DH, Xue X, Li RJ, Hu XX, Guan JY, Tang WX, Xu HE: **[Study on syndromic deafness caused by novel pattern of compound heterozygous variants in the CDH23 gene].** *Zhonghua Er Bi Yan Hou Tou Jing Wai Ke Za Zhi* 2020, **55:**822-829.
33. Loo JC, Liu L, Hao A, Gao L, Agatep R, Shennan M, Summers A, Goldstein AM, Tucker MA, Deters C, et al: **Germline splicing mutations of CDKN2A predispose to melanoma.** *Oncogene* 2003, **22:**6387-6394.
34. Zhang B, Zhang Y, Zhang Y, Liu X, Zhang R, Wang Z, Pan F, Xu N, Shao L: **Identified five variants in CFTR gene that alter RNA splicing by minigene assay.** *Front Genet* 2025, **16:**1543623.

35. Joynt AT, Evans TA, Pellicore MJ, Davis-Marcisak EF, Aksit MA, Eastman AC, Patel SU, Paul KC, Osorio DL, Bowling AD, et al: **Evaluation of both exonic and intronic variants for effects on RNA splicing allows for accurate assessment of the effectiveness of precision therapies.** *PLoS Genet* 2020, **16:**e1009100.
36. Donega S, Rogalska ME, Pianigiani G, Igreja S, Amaral MD, Pagani F: **Rescue of common exon-skipping mutations in cystic fibrosis with modified U1 snRNAs.** *Hum Mutat* 2020, **41:**2143-2154.
37. Fidler MC, Buckley A, Sullivan JC, Statia M, Boj SF, Vries RGJ, Munck A, Higgins M, Moretto Zita M, Negulescu P, et al: **G970R-CFTR Mutation (c.2908G>C) Results Predominantly in a Splicing Defect.** *Clin Transl Sci* 2021, **14:**656-663.
38. Amato F, Scudieri P, Musante I, Tomati V, Caci E, Comegna M, Maietta S, Manzoni F, Di Lullo AM, De Wachter E, et al: **Two CFTR mutations within codon 970 differently impact on the chloride channel functionality.** *Hum Mutat* 2019, **40:**742-748.
39. Carrico JN, Goncalves CI, Aragues JM, Lemos MC: **Kallmann Syndrome: Functional Analysis of a CHD7 Missense Variant Shows Aberrant RNA Splicing.** *Int J Mol Sci* 2024, **25**.
40. Inoue T, Nagano C, Matsuo M, Yamamura T, Sakakibara N, Horinouchi T, Shibagaki Y, Ichikawa D, Aoto Y, Ishiko S, et al: **Functional analysis of suspected splicing variants in CLCN5 gene in Dent disease 1.** *Clin Exp Nephrol* 2020, **24:**606-612.
41. Xin Q, Liu Q, Liu Z, Shi X, Liu X, Zhang R, Hong Y, Zhao X, Shao L: **Twelve exonic variants in the SLC12A1 and CLCNKB genes alter RNA splicing in a minigene assay.** *Frontiers in Genetics* 2022, **13**.
42. Perdomo-Ramirez A, de Armas-Ortiz M, Ramos-Trujillo E, Suarez-Artiles L, Claverie-Martin F: **Exonic CLDN16 mutations associated with familial hypomagnesemia with hypercalciuria and nephrocalcinosis can induce deleterious mRNA alterations.** *BMC Med Genet* 2019, **20:**6.
43. Perdomo-Ramirez A, Aguirre M, Davitaia T, Ariceta G, Ramos-Trujillo E, RenalTube G, Claverie-Martin F: **Characterization of two novel mutations in the claudin-16 and claudin-19 genes that cause familial hypomagnesemia with hypercalciuria and nephrocalcinosis.** *Gene* 2019, **689:**227-234.
44. Rawnsley K, Weisschuh N, Kohl S, Reuter P: **Comprehensive functional splicing analysis of non-canonical CNGB3 variants using in vitro minigene splice assays.** *J Pathol* 2025, **266:**322-336.
45. Booth KT, Ghaffar A, Rashid M, Hovey LT, Hussain M, Frees K, Renkes EM, Nishimura CJ, Shahzad M, Smith RJ, et al: **Novel loss-of-function mutations in COCH cause autosomal recessive nonsyndromic hearing loss.** *Hum Genet* 2020, **139:**1565-1574.
46. Shao Y, Zhang R: **Identifying six single nucleotide variants in the COL17A1 gene that alter RNA splicing: database analysis and minigene assays.** *Sci Rep* 2025, **15:**11387.
47. Li L, Cao Y, Zhao F, Mao B, Ren X, Wang Y, Guan Y, You Y, Li S, Yang T, Zhao X: **Validation and Classification of Atypical Splicing Variants Associated With Osteogenesis Imperfecta.** *Frontiers in Genetics* 2019, **10**.

48. Satoh C, Kondoh T, Shimizu H, Kinoshita A, Mishima H, Nishimura G, Miyazaki M, Okano K, Kumai Y, Yoshiura KI: **Brothers with novel compound heterozygous mutations in COL27A1 causing dental and genital abnormalities.** *Eur J Med Genet* 2021, **64:**104125.
49. Deng H, Zhang Y, Ding J, Wang F: **Presumed COL4A3/COL4A4 Missense/Synonymous Variants Induce Aberrant Splicing.** *Front Med (Lausanne)* 2022, **9:**838983.
50. Daga S, Loberti L, Rollo G, Adamo L, Colavecchio OL, Brunelli G, Zguro K, Tripodi SA, Guarnieri A, Garosi G, et al: **Slowly progressive autosomal dominant Alport Syndrome due to COL4A3 splicing variant.** *Eur J Hum Genet* 2025, **33:**461-467.
51. Rossanti R, Horinouchi T, Yamamura T, Nagano C, Sakakibara N, Ishiko S, Aoto Y, Kondo A, Nagai S, Okada E, et al: **Evaluation of Suspected Autosomal Alport Syndrome Synonymous Variants.** *Kidney360* 2022, **3:**497-505.
52. Aoto Y, Horinouchi T, Yamamura T, Kondo A, Nagai S, Ishiko S, Okada E, Rossanti R, Sakakibara N, Nagano C, et al: **Last Nucleotide Substitutions of COL4A5 Exons Cause Aberrant Splicing.** *Kidney Int Rep* 2022, **7:**108-116.
53. Okada E, Aoto Y, Horinouchi T, Yamamura T, Ichikawa Y, Tanaka Y, Ueda C, Kitakado H, Kondo A, Sakakibara N, et al: **Aberrant splicing caused by exonic single nucleotide variants positioned 2nd or 3rd to the last nucleotide in the COL4A5 gene.** *Clin Exp Nephrol* 2023, **27:**218-226.
54. Zhang R, Lang Y, Shi X, Zhang Y, Liu X, Pan F, Qiao D, Teng X, Shao L: **Three exonic variants in the COL4A5 gene alter RNA splicing in a minigene assay.** *Mol Genet Genomic Med* 2024, **12:**e2395.
55. Ma N, Zhu Z, Liu J, Peng Y, Zhao X, Tang W, Jia Z, Xi H, Gao B, Wang H, Du J: **Clinical and genetic analysis of classical Ehlers-Danlos syndrome patient caused by synonymous mutation in COL5A2.** *Mol Genet Genomic Med* 2021, **9:**e1632.
56. Bellingrath J-S, McClements ME, Fischer MD, MacLaren RE: **Development of a functional assay for the assessment of two common CRB1 mutations.** *Investigative Ophthalmology & Visual Science* 2022, **63:**1766 – F0315-1766 – F0315.
57. Zou M, Guven A, Binessa HA, Al-Rijjal RA, Meyer BF, Alzahrani AS, Shi Y: **Molecular Analysis of CYP27B1 Mutations in Vitamin D-Dependent Rickets Type 1A: c.590G > A (p.G197D) Missense Mutation Causes a RNA Splicing Error.** *Frontiers in Genetics* 2020, **11**.
58. Zou M, Guven A, BinEssa HA, Al-Rijjal RA, Meyer BF, Alzahrani AS, Shi Y: **Molecular Analysis of CYP27B1 Mutations in Vitamin D-Dependent Rickets Type 1A: c.590G > A (p.G197D) Missense Mutation Causes a RNA Splicing Error.** *Front Genet* 2020, **11:**607517.
59. Brodehl A, Hain C, Flottmann F, Ratnavadivel S, Gaertner A, Klauke B, Kalinowski J, Korperich H, Gummert J, Paluszkiewicz L, et al: **The Desmin Mutation DES-c.735G>C Causes Severe Restrictive Cardiomyopathy by Inducing In-Frame Skipping of Exon-3.** *Biomedicines* 2021, **9**.
60. Lee KE, Lee SK, Jung SE, Lee Z, Kim JW: **Functional splicing assay of DSPP mutations in hereditary dentin defects.** *Oral Dis* 2011, **17:**690-695.

61. Kergourlay V, Raï G, Blandin G, Salgado D, Béroud C, Lévy N, Krahn M, Bartoli M: **Identification of Splicing Defects Caused by Mutations in the Dysferlin Gene.** *Human Mutation* 2014, **35:**1532-1541.
62. Thomas HB, Wood KA, Buczek WA, Gordon CT, Pingault V, Attié-Bitach T, Hentges KE, Varghese VC, Amiel J, Newman WG, O'Keefe RT: **<i>EFTUD2</i> missense variants disrupt protein function and splicing in mandibulofacial dysostosis Guion-Almeida type.** *Human Mutation* 2020, **41:**1372-1382.
63. Jacob A, Pasquier J, Carapito R, Auradé F, Molitor A, Froguel P, Fakhro K, Halabi N, Viot G, Bahram S, Rafii A: **A de novo synonymous variant in EFTUD2 disrupts normal splicing and causes mandibulofacial dysostosis with microcephaly: case report.** *BMC Medical Genetics* 2020, **21**.
64. Vojcek E, Keszthelyi TM, Javorszky E, Balogh L, Tory K: **EPG5 c.1007A > G mutation in a sibling pair with rapidly progressing Vici syndrome.** *Ann Hum Genet* 2020, **84:**80-86.
65. Morin M, Borreguero L, Booth KT, Lachgar M, Huygen P, Villamar M, Mayo F, Barrio LC, Santos Serrao de Castro L, Morales C, et al: **Insights into the pathophysiology of DFNA10 hearing loss associated with novel EYA4 variants.** *Sci Rep* 2020, **10:**6213.
66. Chase A, Score J, Lin F, Bryant C, Waghorn K, Yapp S, Carreno-Tarragona G, Aranaz P, Villasante A, Ernst T, Cross NCP: **Mutational mechanisms of EZH2 inactivation in myeloid neoplasms.** *Leukemia* 2020, **34:**3206-3214.
67. Paraboschi EM, Menegatti M, Peyvandi F, Duga S, Asselta R: **Understanding the Impact of Aberrant Splicing in Coagulation Factor V Deficiency.** *Int J Mol Sci* 2019, **20**.
68. Balestra D, Maestri I, Branchini A, Ferrarese M, Bernardi F, Pinotti M: **An Altered Splicing Registry Explains the Differential ExSpeU1-Mediated Rescue of Splicing Mutations Causing Haemophilia A.** *Front Genet* 2019, **10:**974.
69. Jourdy Y, Fretigny M, Nougier C, Negrier C, Bozon D, Vinciguerra C: **Splicing analysis of 26 F8 nucleotide variations using a minigene assay.** *Haemophilia* 2019, **25:**306-315.
70. Fama R, Borroni E, Zanolini D, Merlin S, Bruscaggin V, Walker GE, Olgasi C, Babu D, Agnelli Giacchello J, Valeri F, et al: **Identification and functional characterization of a novel splicing variant in the F8 coagulation gene causing severe hemophilia A.** *J Thromb Haemost* 2020, **18:**1050-1064.
71. Odaira K, Tamura S, Suzuki N, Kakihara M, Hattori Y, Tokoro M, Suzuki S, Takagi A, Katsumi A, Hayakawa F, et al: **Apparent synonymous mutation F9 c.87A>G causes secretion failure by in-frame mutation with aberrant splicing.** *Thromb Res* 2019, **179:**95-103.
72. Dreumont N, Poudrier JA, Bergeron A, Levy HL, Baklouti F, Tanguay RM: **A missense mutation (Q279R) in the fumarylacetoacetate hydrolase gene, responsible for hereditary tyrosinemia, acts as a splicing mutation.** *BMC Genet* 2001, **2:**9.

73. Donovan FX, Solanki A, Mori M, Chavan N, George M, C SK, Okuno Y, Muramastsu H, Yoshida K, Shimamoto A, et al: **A founder variant in the South Asian population leads to a high prevalence of FANCL Fanconi anemia cases in India.** *Hum Mutat* 2020, **41:**122-128.
74. Wang X: **Molecular characterization of a novel defect occurring de novo associated with erythropoietic protoporphyria.** *Biochim Biophys Acta* 1996, **1316:**149-152.
75. Carion N, Briand A, Cuisset L, Pacot L, Afenjar A, Bienvenu T: **Loss of the KH1 domain of FMR1 in humans due to a synonymous variant causes global developmental retardation.** *Gene* 2020, **753:**144793.
76. Bergsma AJ, In 't Groen SLM, Catalano F, Yamanaka M, Takahashi S, Okumiya T, van der Ploeg AT, Pijnappel W: **A generic assay for the identification of splicing variants that induce nonsense-mediated decay in Pompe disease.** *Eur J Hum Genet* 2021, **29:**422-433.
77. Crespo C, Eiroa H, Otegui MI, Bonetto MC, Chertkoff L, Gravina LP: **Molecular analysis of GALT gene in Argentinian population: Correlation with enzyme activity and characterization of a novel Duarte-like allele.** *Mol Genet Metab Rep* 2020, **25:**100695.
78. Bouvet D, Blondel A, De Sainte Agathe J-M, Leroy G, Saint-Martin C, Bellanné-Chantelot C: **Evaluation in Monogenic Diabetes of the Impact of GCK, HNF1A, and HNF4A Variants on Splicing through the Combined Use of In Silico Tools and Minigene Assays.** *Human Mutation* 2023, **2023:**1-13.
79. Tiulpakov A, Zubkova N, Makretskaya N, Krasnova TS, Melnikova AI, Fedyaeva AS, Vasilyev E, Petrov VM, Rubtsov PM: **Minigene splicing assessment of 20 novel synonymous and intronic glucokinase gene variants identified in patients with maturity-onset diabetes of the young.** *Hum Mutat* 2020, **41:**129-132.
80. Sommerville EW, Dalla Rosa I, Rosenberg MM, Bruni F, Thompson K, Rocha M, Blakely EL, He L, Falkous G, Schaefer AM, et al: **Identification of a novel heterozygous guanosine monophosphate reductase (GMPR) variant in a patient with a late-onset disorder of mitochondrial DNA maintenance.** *Clin Genet* 2020, **97:**276-286.
81. Zhe J, Chen S, Chen X, Liu Y, Li Y, Zhou X, Zhang J: **A novel heterozygous splice-altering mutation in HFM1 may be a cause of premature ovarian insufficiency.** *J Ovarian Res* 2019, **12:**61.
82. Levy-Khademi F, Zeligson S, Lavi E, Klopstock T, Chertin B, Avnon-Ziv C, Abulibdeh A, Renbaum P, Rosen T, Perlberg-Bengio S, et al: **The novel founder homozygous V225M mutation in the HSD17B3 gene causes aberrant splicing and XY-DSD.** *Endocrine* 2020, **69:**650-654.
83. Lorès P, Kherraf Z-E, Amiri-Yekta A, Whitfield M, Daneshipour A, Stouvenel L, Cazin C, Cavarocchi E, Coutton C, Llabador M-A, et al: **A missense mutation in IFT74, encoding for an essential component for intraflagellar transport of Tubulin, causes asthenozoospermia and male infertility without clinical signs of Bardet–Biedl syndrome.** *Human Genetics* 2021, **140:**1031-1043.

84. Lores P, Kherraf ZE, Amiri-Yekta A, Whitfield M, Daneshipour A, Stouvenel L, Cazin C, Cavarocchi E, Coutton C, Llabador MA, et al: **A missense mutation in IFT74, encoding for an essential component for intraflagellar transport of Tubulin, causes asthenozoospermia and male infertility without clinical signs of Bardet-Biedl syndrome.** *Hum Genet* 2021, **140:**1031-1043.
85. Kanai N, Yanai F, Hirose S, Nibu K, Izuhara K, Tani T, Kubota T, Mitsudome A: **A G to A transition at the last nucleotide of exon 6 of the gamma c gene (868G-->A) may result in either a splice or missense mutation in patients with X-linked severe combined immunodeficiency.** *Hum Genet* 1999, **104:**36-42.
86. Keefer-Jacques E, Valente N, Jacko AM, Matwijec G, Reese A, Tekriwal A, Loomes KM, Spinner NB, Gilbert MA: **Investigation of cryptic JAG1 splice variants as a cause of Alagille syndrome and performance evaluation of splice predictor tools.** *HGG Adv* 2024, **5:**100351.
87. Wang X, Tian R, Zhang H, Abdalla M, Bai L, Lv Y, Gao M, Lin G, Liu Q, Liu Y, et al: **Combination of Synonymous and Missense Mutations in JAK3 Gene Contributes to Severe Combined Immunodeficiency in One Child.** *Human Mutation* 2023, **2023:**1-9.
88. Cocciadiferro D, Agolini E, Digilio MC, Sinibaldi L, Castori M, Silvestri E, Dotta A, Dallapiccola B, Novelli A: **The splice c.1815G>A variant in KIAA0586 results in a phenotype bridging short-rib-polydactyly and oral-facial-digital syndrome: A case report and literature review.** *Medicine (Baltimore)* 2020, **99:**e19169.
89. Guo Z, Huo X, Wu D, Hao B, Liao S: **A Novel Variant of the KIF11 Gene, c.2922G>T, Is Associated with Microcephaly by Affecting RNA Splicing.** *Dev Neurosci* 2022, **44:**113-120.
90. Mittwollen R, Wohlfart S, Park J, Grosch E, Has C, Hohenester E, Schneider H, Hammersen J: **Aberrant splicing as potential modifier of the phenotype of junctional epidermolysis bullosa.** *J Eur Acad Dermatol Venereol* 2020, **34:**2127-2134.
91. Ramprasad VL, Soumittra N, Nancarrow D, Sen P, McKibbin M, Williams GA, Arokiasamy T, Lakshmipathy P, Inglehearn CF, Kumaramanickavel G: **Identification of a novel splice-site mutation in the Lebercilin (LCA5) gene causing Leber congenital amaurosis.** *Mol Vis* 2008, **14:**481-486.
92. Kato K, Ohno S, Sonoda K, Fukuyama M, Makiyama T, Ozawa T, Horie M: **<i>LMNA</i> Missense Mutation Causes Nonsense-Mediated mRNA Decay and Severe Dilated Cardiomyopathy.** *Circulation: Genomic and Precision Medicine* 2020, **13:**435-443.
93. Kato K, Ohno S, Sonoda K, Fukuyama M, Makiyama T, Ozawa T, Horie M: **LMNA Missense Mutation Causes Nonsense-Mediated mRNA Decay and Severe Dilated Cardiomyopathy.** *Circ Genom Precis Med* 2020, **13:**435-443.
94. Bhuyan F, de Jesus AA, Mitchell J, Leikina E, VanTries R, Herzog R, Onel KB, Oler A, Montealegre Sanchez GA, Johnson KA, et al: **Novel Majeed Syndrome-Causing LPIN2 Mutations Link Bone Inflammation to Inflammatory M2 Macrophages and Accelerated Osteoclastogenesis.** *Arthritis Rheumatol* 2021, **73:**1021-1032.

95. Howaldt A, Hennig AF, Rolvien T, Rossler U, Stelzer N, Knaus A, Bottger S, Zustin J, Geissler S, Oheim R, et al: **Adult Osteosclerotic Metaphyseal Dysplasia With Progressive Osteonecrosis of the Jaws and Abnormal Bone Resorption Pattern Due to a LRRK1 Splice Site Mutation.** *J Bone Miner Res* 2020, **35:**1322-1332.
96. Caburet S, Todeschini AL, Petrillo C, Martini E, Farran ND, Legois B, Livera G, Younis JS, Shalev S, Veitia RA: **A truncating MEIOB mutation responsible for familial primary ovarian insufficiency abolishes its interaction with its partner SPATA22 and their recruitment to DNA double-strand breaks.** *EBioMedicine* 2019, **42:**524-531.
97. Pagenstecher C, Wehner M, Friedl W, Rahner N, Aretz S, Friedrichs N, Sengteller M, Henn W, Buettner R, Propping P, Mangold E: **Aberrant splicing in MLH1 and MSH2 due to exonic and intronic variants.** *Hum Genet* 2006, **119:**9-22.
98. Auclair J, Busine MP, Navarro C, Ruano E, Montmain G, Desseigne F, Saurin JC, Lasset C, Bonadona V, Giraud S, et al: **Systematic mRNA analysis for the effect ofMLH1 andMSH2 missense and silent mutations on aberrant splicing.** *Human Mutation* 2006, **27:**145-154.
99. van der Klift HM, Jansen AM, van der Steenstraten N, Bik EC, Tops CM, Devilee P, Wijnen JT: **Splicing analysis for exonic and intronic mismatch repair gene variants associated with Lynch syndrome confirms high concordance between minigene assays and patient RNA analyses.** *Mol Genet Genomic Med* 2015, **3:**327-345.
100. Jansen AM, van der Klift HM, Roos MA, van Eendenburg JD, Tops CM, Wijnen JT, Hes FJ, Morreau H, van Wezel T: **RNA analysis of cancer predisposing genes in formalin-fixed paraffin-embedded tissue determines aberrant splicing.** *Eur J Hum Genet* 2018, **26:**1143-1150.
101. Vettore S, De Rocco D, Gerber B, Scandellari R, Bianco AM, Balduini CL, Pecci A, Fabris F, Savoia A: **A G to C transversion at the last nucleotide of exon 25 of the MYH9 gene results in a missense mutation rather than in a splicing defect.** *Eur J Med Genet* 2010, **53:**256-260.
102. Xiang Y, Xu C, Xu Y, Zhou L, Tang S, Xu X: **Novel compound heterozygous synonymous and missense variants in the <i>MYO7A</i> gene identified by next-generation sequencing in a Chinese family with nonsyndromic hearing loss.** *Journal of Clinical Laboratory Analysis* 2022, **36**.
103. Li N, Xu Y, Yu T, Yao R, Chen J, Luo C, Wang J: **Further delineation of bone marrow failure syndrome caused by novel compound heterozygous variants of MYSM1.** *Gene* 2020, **757:**144938.
104. Roth IL, Salamon P, Freund T, Gadot YB, Baron S, Hershkovitz T, Shefler I, Hanna S, Confino-Cohen R, Bentur L, Hagin D: **Novel NCF2 Mutation Causing Chronic Granulomatous Disease.** *J Clin Immunol* 2020, **40:**977-986.
105. Koczkowska M, Callens T, Chen Y, Gomes A, Hicks AD, Sharp A, Johns E, Uhas KA, Armstrong L, Bosanko KA, et al: **Clinical spectrum of individuals with pathogenic NF1 missense variants affecting p.Met1149, p.Arg1276, and p.Lys1423: genotype-phenotype study in neurofibromatosis type 1.** *Hum Mutat* 2020, **41:**299-315.

106. Ars E, Serra E, Garcia J, Kruyer H, Gaona A, Lazaro C, Estivill X: **Mutations affecting mRNA splicing are the most common molecular defects in patients with neurofibromatosis type 1.** *Hum Mol Genet* 2000, **9:**237-247.
107. Qiao F, Zhang C, Wang Y, Liu G, Shao B, Hu P, Xu Z: **Case Report: Prenatal Whole-Exome Sequencing to Identify a Novel Heterozygous Synonymous Variant in NIPBL in a Fetus With Cornelia de Lange Syndrome.** *Front Genet* 2021, **12:**628890.
108. Bychkov I, Filatova A, Perelman G, Proshlyakova T, Korotkova D, Klyushnikov S, Karpova M, Tabakov V, Baydakova G, Ilyushkina A, et al: **Additive effect of frequent polymorphism and rare synonymous variant alters splicing in twin patients with Niemann-Pick disease type C.** *Eur J Hum Genet* 2022, **30:**133-136.
109. Yu B, Gao Y, Mao J, Wang X, Nie M, Wu X: **Mutation of c.244G>T in NR5A1 gene causing 46, XY DSD by affecting RNA splicing.** *Orphanet J Rare Dis* 2021, **16:**370.
110. Balestra D, Ferrarese M, Lombardi S, Ziliotto N, Branchini A, Petersen N, Bosma P, Pinotti M, van de Graaf SFJ: **An Exon-Specific Small Nuclear U1 RNA (ExSpeU1) Improves Hepatic OTC Expression in a Splicing-Defective spf/ash Mouse Model of Ornithine Transcarbamylase Deficiency.** *Int J Mol Sci* 2020, **21**.
111. Valenzuela-Palomo A, Bueno-Martinez E, Sanoguera-Miralles L, Lorca V, Fraile-Bethencourt E, Esteban-Sanchez A, Gomez-Barrero S, Carvalho S, Allen J, Garcia-Alvarez A, et al: **Splicing predictions, minigene analyses, and ACMG-AMP clinical classification of 42 germline PALB2 splice-site variants.** *J Pathol* 2022, **256:**321-334.
112. Li H, Lin Y, Ma W, Yu T, Dong L, Chen Y, Fan S, Luo G, Zhang J, Song G: **Identification of a PATL2 missense variant (c.877G>T) disrupting canonical splicing and contributing to female infertility.** *Front Genet* 2025, **16:**1611138.
113. Davydenko K, Filatova A, Skoblov M: **Assessing Splicing Variants in the PAX6 Gene: A Comprehensive Minigene Approach.** *J Cell Mol Med* 2025, **29:**e70459.
114. Filatova AY, Vasilyeva TA, Marakhonov AV, Voskresenskaya AA, Zinchenko RA, Skoblov MY: **Functional reassessment of PAX6 single nucleotide variants by in vitro splicing assay.** *Eur J Hum Genet* 2019, **27:**488-493.
115. Garcia-Solaesa V, Serrano-Lorenzo P, Ramos-Arroyo MA, Blazquez A, Pagola-Lorz I, Artigas-Lopez M, Arenas J, Martin MA, Jerico-Pascual I: **A Novel Missense Variant Associated with A Splicing Defect in A Myopathic Form of PGK1 Deficiency in The Spanish Population.** *Genes (Basel)* 2019, **10**.
116. Pan F, Zhang R, Liu X, Shi X, Xin Q, Qiao D, Li C, Zhang Y, Chen M, Guo W, et al: **Three exonic variants in the PHEX gene cause aberrant splicing in a minigene assay.** *Front Genet* 2024, **15:**1353674.
117. Gavilan CM, Murata-Nakamura Y, Porter R, Cutler B, Rai S, Kim HG, Iwase S: **Molecular Consequences of a Missense PHF21A Variant, c.1285G > A, Associated With Syndromic Neurodevelopmental Disorder.** *Cell Mol Neurobiol* 2025, **45:**62.
118. Gonzalez-Paredes FJ, Ramos-Trujillo E, Claverie-Martin F: **Defective pre-mRNA splicing in PKD1 due to presumed missense and synonymous mutations causing autosomal dominant polycystic disease.** *Gene* 2014, **546:**243-249.

119. Deng LX, Yang Y, Yang J, Zhou LW, Wang K, Zhou JH: **A Presumed Synonymous Mutation of PKD2 Caused Autosomal Dominant Polycystic Kidney Disease in a Chinese Family.** *Curr Med Sci* 2021, **41:**1029-1036.
120. Neuser S, Krey I, Schwan A, Abou Jamra R, Bartolomaeus T, Doring J, Syrbe S, Plassmann M, Rohde S, Roth C, et al: **Prenatal phenotype of PNKP-related primary microcephaly associated with variants affecting both the FHA and phosphatase domain.** *Eur J Hum Genet* 2022, **30:**101-110.
121. Lessel D, Rading K, Campbell SE, Thiele H, Altmuller J, Gordon LB, Kubisch C: **A novel homozygous synonymous variant further expands the phenotypic spectrum of POLR3A-related pathologies.** *Am J Med Genet A* 2022, **188:**216-223.
122. Chen XY, Song DY, Jiang L, Tan DD, Liu YD, Liu JY, Chang XZ, Xing GG, Toda T, Xiong H: **Phenotype and Genotype Study of Chinese POMT2-Related alpha-Dystroglycanopathy.** *Front Genet* 2021, **12:**692479.
123. Potrony M, Puig-Butille JA, Ribera-Sola M, Iyer V, Robles-Espinoza CD, Aguilera P, Carrera C, Malvehy J, Badenas C, Landi MT, et al: **POT1 germline mutations but not TERT promoter mutations are implicated in melanoma susceptibility in a large cohort of Spanish melanoma families.** *Br J Dermatol* 2019, **181:**105-113.
124. Gergics P, Smith C, Bando H, Jorge AAL, Rockstroh-Lippold D, Vishnopolska SA, Castinetti F, Maksutova M, Carvalho LRS, Hoppmann J, et al: **High-throughput splicing assays identify missense and silent splice-disruptive POU1F1 variants underlying pituitary hormone deficiency.** *Am J Hum Genet* 2021, **108:**1526-1539.
125. Cho SY, Lau EY, Luk DC, Law CY, Lai CK, Lam CW: **Novel PPOX exonic mutation inducing aberrant splicing in a patient with homozygous variegate porphyria.** *Clin Chim Acta* 2021, **512:**117-120.
126. Ragamin A, Yigit G, Bousset K, Beleggia F, Verheijen FW, de Wit MY, Strom TM, Dork T, Wollnik B, Mancini GMS: **Human RAD50 deficiency: Confirmation of a distinctive phenotype.** *Am J Med Genet A* 2020, **182:**1378-1386.
127. Yang C, Arnold AG, Catchings A, Rai V, Stadler ZK, Zhang L: **The RAD51D c.82G>A (p.Val28Met) variant disrupts normal splicing and is associated with hereditary ovarian cancer.** *Breast Cancer Res Treat* 2021, **185:**869-877.
128. Fichou Y, Gehannin P, Corre M, Le Guern A, Le Marechal C, Le Gac G, Ferec C: **Extensive functional analyses of RHD splice site variants: Insights into the potential role of splicing in the physiology of Rh.** *Transfusion* 2015, **55:**1432-1443.
129. Raud L, Ka C, Gourlaouen I, Callebaut I, Ferec C, Le Gac G, Fichou Y: **Functional analysis of novel RHD variants: splicing disruption is likely to be a common mechanism of variant D phenotype.** *Transfusion* 2019, **59:**1367-1375.
130. Zhong Z, Rong F, Dai Y, Yibulayin A, Zeng L, Liao J, Wang L, Huang Z, Zhou Z, Chen J: **Seven novel variants expand the spectrum of RPE65-related Leber congenital amaurosis in the Chinese population.** *Mol Vis* 2019, **25:**204-214.
131. Demirci FY, Radak AL, Rigatti BW, Mah TS, Gorin MB: **A presumed missense mutation of RPGR causes abnormal RNA splicing with exon skipping.** *Am J Ophthalmol* 2004, **138:**504-505.
132. Sparber P, Mikhaylova S, Galkina V, Itkis Y, Skoblov M: **Case Report: Functional Investigation of an Undescribed Missense Variant Affecting Splicing in a Patient With Dravet Syndrome.** *Frontiers in Neurology* 2021, **12**.

133. Escobar H, Krause A, Keiper S, Kieshauer J, Muthel S, de Paredes MG, Metzler E, Kuhn R, Heyd F, Spuler S: **Base editing repairs an SGCA mutation in human primary muscle stem cells.** *JCI Insight* 2021, **6**.
134. Shi X, Wang H, Zhang R, Liu Z, Guo W, Wang S, Liu X, Lang Y, Bottillo I, Dong B, Shao L: **Minigene splicing assays reveal new insights into exonic variants of the <i>SLC12A3</i> gene in Gitelman syndrome.** *Molecular Genetics & Genomic Medicine* 2023, **11**.
135. Le Tertre M, Ka C, Raud L, Berlivet I, Gourlaouen I, Richard G, Uguen K, Chen JM, Ferec C, Fichou Y, Le Gac G: **Splicing analysis of SLC40A1 missense variations and contribution to hemochromatosis type 4 phenotypes.** *Blood Cells Mol Dis* 2021, **87:**102527.
136. Wang S, Wang Y, Wang J, Liu Z, Zhang R, Shi X, Han Y, Guo W, Bottillo I, Shao L: **Six Exonic Variants in the SLC5A2 Gene Cause Exon Skipping in a Minigene Assay.** *Front Genet* 2020, **11:**585064.
137. Bai J, Qu Y, Song F, Cao Y, Cheng M, Wang J, Jin Y, Wang H: **Dual Mechanism of a New SMN1 Variant (c.835G>C, p.Gly279Arg) by Interrupting Exon 7 Skipping and YG Oligomerization in Causation of Spinal Muscular Atrophy.** *J Mol Neurosci* 2021, **71:**112-121.
138. Wu H, Lin JH, Tang XY, Marenne G, Zou WB, Schutz S, Masson E, Genin E, Fichou Y, Le Gac G, et al: **Combining full-length gene assay and SpliceAI to interpret the splicing impact of all possible SPINK1 coding variants.** *Hum Genomics* 2024, **18:**21.
139. Zirngibl RA, Wang A, Yao Y, Manolson MF, Krueger J, Dupuis L, Mendoza-Londono R, Voronov I: **Novel c.G630A TCIRG1 mutation causes aberrant splicing resulting in an unusually mild form of autosomal recessive osteopetrosis.** *J Cell Biochem* 2019, **120:**17180-17193.
140. Wang L, Zhang D, Fan C, Zhou X, Liu Z, Zheng B, Zhu L, Jin Y: **Novel Compound Heterozygous TMPRSS15 Gene Variants Cause Enterokinase Deficiency.** *Front Genet* 2020, **11:**538778.
141. Zhou F, Zhong H, Wu B, Cui Y, Li J, Jia X, Yu C, Li D, Shu J, Cai C: **Identification of the synonymous variant c.3141G > A in TNRC6B gene that altered RNA splicing by minigene assay.** *Mol Biol Rep* 2024, **51:**899.
142. Kaissarian NM, Meyer D, Kimchi-Sarfaty C: **Synonymous Variants: Necessary Nuance in Our Understanding of Cancer Drivers and Treatment Outcomes.** *J Natl Cancer Inst* 2022, **114:**1072-1094.
143. Supek F, Minana B, Valcarcel J, Gabaldon T, Lehner B: **Synonymous mutations frequently act as driver mutations in human cancers.** *Cell* 2014, **156:**1324-1335.
144. Bhagavatula G, Rich MS, Young DL, Marin M, Fields S: **A Massively Parallel Fluorescence Assay to Characterize the Effects of Synonymous Mutations on TP53 Expression.** *Mol Cancer Res* 2017, **15:**1301-1307.
145. Austin F, Oyarbide U, Massey G, Grimes M, Corey SJ: **Synonymous mutation in TP53 results in a cryptic splice site affecting its DNA-binding site in an adolescent with two primary sarcomas.** *Pediatr Blood Cancer* 2017, **64**.

146. Pinto EM, Maxwell KN, Halalsheh H, Phillips A, Powers J, MacFarland S, Walsh MF, Breen K, Formiga MN, Kriwacki R, et al: **Clinical and Functional Significance of TP53 Exon 4-Intron 4 Splice Junction Variants.** *Mol Cancer Res* 2022, **20:**207-216.
147. Shiraishi Y, Kataoka K, Chiba K, Okada A, Kogure Y, Tanaka H, Ogawa S, Miyano S: **A comprehensive characterization of cis-acting splicing-associated variants in human cancer.** *Genome Res* 2018, **28:**1111-1125.
148. Li X, Sun Y, Gong L, Zheng L, Chen K, Zhou Y, Gu Y, Xu Y, Guo Q, Hong Z, et al: **A novel homozygous mutation in TREM2 found in a Chinese early-onset dementia family with mild bone involvement.** *Neurobiol Aging* 2020, **86:**201 e201-201 e207.
149. Goyal S, Vanita V: **A missense mutation in TTC8/BBS8 affecting mRNA splicing in patients with non-syndromic retinitis pigmentosa.** *Molecular Genetics and Genomics* 2022, **297:**1439-1449.
150. Wang X, You B, Yin F, Chen C, He H, Liu F, Pan Z, Ni X, Pang N, Peng J: **A presumed missense variant in the U2AF2 gene causes exon skipping in neurodevelopmental diseases.** *J Hum Genet* 2023, **68:**375-382.
151. Domingo D, Nawaz U, Corbett M, Espinoza JL, Tatton-Brown K, Coman D, Wilkinson MF, Gecz J, Jolly LA: **A synonymous UPF3B variant causing a speech disorder implicates NMD as a regulator of neurodevelopmental disorder gene networks.** *Hum Mol Genet* 2020, **29:**2568-2578.
152. Borràs N, Orriols G, Batlle J, Pérez-Rodríguez A, Fidalgo T, Martinho P, López-Fernández MF, Rodríguez-Trillo Á, Lourés E, Parra R, et al: **Unraveling the effect of silent, intronic and missense mutations on <i>VWF</i> splicing: contribution of next generation sequencing in the study of mRNA.** *Haematologica* 2019, **104:**587-598.
